# Discovery and Photoisomerization of New Pyrrolosesquiterpenoids Glaciapyrroles D and E, from Deep-Sea Sediment *Streptomyces* sp.

**DOI:** 10.3390/md20050281

**Published:** 2022-04-22

**Authors:** Keebeom Ko, Seong-Hwan Kim, Subin Park, Hwa Seung Han, Jae Kyun Lee, Jin Wook Cha, Sunghoon Hwang, Ki Young Choi, Yoon-Jae Song, Sang-Jip Nam, Jongheon Shin, Seung-Il Nam, Hak Cheol Kwon, Jin-Soo Park, Dong-Chan Oh

**Affiliations:** 1 Natural Products Research Institute, College of Pharmacy, Seoul National University, 1 Gwanak-ro, Gwanak-gu, Seoul 08826, Korea; gogiup0218@snu.ac.kr (K.K.); sunghooi@snu.ac.kr (S.H.); shinj@snu.ac.kr (J.S.); 2 Natural Product Informatics Research Center, Korea Institute of Science and Technology (KIST), Gangneung Institute, Gangneung 25451, Korea; seonghwankim@kist.re.kr (S.-H.K.); hanhwaseung@kist.re.kr (H.S.H.); elmarit@kist.re.kr (J.W.C.); kiyoungchoi@kist.re.kr (K.Y.C.); hkwon@kist.re.kr (H.C.K.); 3 Department of Life Science, Gachon University, Seongnam-daero 1342, Sujeong-gu, Seongnam 13120, Korea; tnqls8634@gachon.ac.kr (S.P.); songyj@gachon.ac.kr (Y.-J.S.); 4 Neuro-Medicine, Korea Institute of Science and Technology, Hwarang-ro 14-gil 5, Seongbuk-gu, Seoul 02792, Korea; j9601@kist.re.kr; 5 Department of Chemistry and Nanoscience, Ewha Womans University, Seoul 03760, Korea; sjnam@ewha.ac.kr; 6 Division of Glacial Environment Research, Korea Polar Research Institute, Incheon 21990, Korea; sinam@kopri.re.kr

**Keywords:** *Streptomyces*, glaciapyrrole, deep-sea sediment, structure elucidation, photoisomerization, pyrrolosesquiterpene

## Abstract

Two new pyrrolosesquiterpenes, glaciapyrroles D (**1**) and E (**2**) were discovered along with the previously reported glaciapyrrole A (**3**) from *Streptomyces* sp. GGS53 strain isolated from deep-sea sediment. This study elucidated the planar structures of **1** and **2** using nuclear magnetic resonance (NMR), mass spectrometry (MS), ultraviolet (UV), and infrared (IR) spectroscopic data. The absolute configurations of the glaciapyrroles were determined by Mosher’s method, circular dichroism spectroscopy, and X-ray crystallography. Under 366 nm UV irradiation, the glaciapyrroles were systematically converted to the corresponding photoglaciapyrroles (**4**–**6**) via photoisomerization, resulting in the diversification of the glaciapyrrole family compounds. The transformation of the glaciapyrrole *Z* to *E* isomers occurred in a 1:1 ratio, based on virtual validation of the photoisomerization of these olefinic compounds by ^1^H-NMR spectroscopy and liquid chromatography/mass spectrometry (LC/MS) analysis. Finally, when encapsulated in poly(lactic-co-glycolic acid) nanoparticles, glaciapyrrole E and photoglaciapyrrole E displayed significant inhibitory activity against influenza A virus. This is the first report of antiviral effects from glaciapyrrole family compounds, whose biological functions have only been subjected to limited studies so far.

## 1. Introduction

Over the past decades, marine natural products have been considered prolific sources of bioactive compounds with structural diversity [1,2]. Since the discovery of salinosporamide A, a drug candidate for treating myeloma, from marine actinomycete (*Salinispora tropica*), there has been active investigation into the chemistry of actinobacteria in marine habitats [3]. However, because of their limited accessibility, research on actinomycetes from the deep sea is rare, even though these bacteria are expected to possess unique metabolism due to their environment where no light penetrates, and enormous water column pressure exists [4]. To address this issue, we collected sediment samples with a multi-corer from the deep-sea floor (over 2000 m depth) in the East Sea of Korea, and isolated more than 70 actinobacterial strains. Subsequent chemical profiling of the strains based on liquid chromatography/mass spectrometry (LC/MS) revealed that *Streptomyces* sp. GGS53 produced new pyrrole-bearing sesquiterpenoids, glaciapyrroles D and E (**1** and **2**), along with the previously reported glaciapyrrole A (**3**).

**Figure 1:**
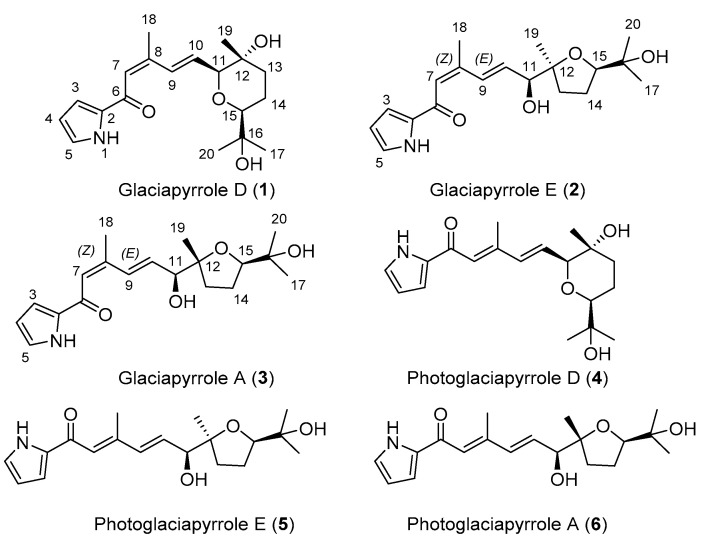
Chemical structures of glaciapyrrole D, E, and A; photoglaciapyrrole D, E, and A (**1**–**6**).

During the purification processes for the glaciapyrroles under laboratory conditions, we found that these compounds were slowly degraded to their geometric isomers due to unavoidable exposure to light, which would not occur in the natural deep-sea environment. In pharmacology, the activity of an organic compound is often altered by external stimuli such as non-ionizing light [5]. As examples from natural products, UV radiation on phytochemicals, such as resveratrol, licochalcone A, and tetramethoxystilbene, induced double-bond isomerization to expand their structural diversity in several previous reports [6,7,8]. Therefore, the observed isomerization phenomenon inspired us to apply artificial photoisomerization to diversify the structures of the glaciapyrroles. In a recent study, we photochemically modified the structures of tropolone-bearing sesquiterpenes juniperone A and norjuniperone A and systematically generated new compounds [9]. By analyzing the conversion of the glaciapyrroles through LC/MS connected with a post-column reactor coil and UV source in the present study, we found that the glaciapyrroles were systematically converted to new derivatives, named photoglaciapyrroles (PG), by *E*/*Z* isomerization of their double bonds and subsequently focused on characterizing the structures of the new members of the glaciapyrrole family (**1** and **2**) and their derivatives by photoirradiation, photoglaciapyrroles D and E (**4** and **5**) (Figure 1). In addition, the biological activity of the glaciapyrrole family has been understudied despite their unique structural features, and only weak cytotoxicity against a few cancer cell lines have been reported. Here, the glaciapyrroles and photoglaciapyrroles were evaluated in viral RNA and plaque reduction assays against influenza A virus (IAV) to determine their antiviral activities. Overall, this paper reports the discovery of new members of the glaciapyrrole family, their systematic conversion to photoglaciapyrroles by photoisomerization, and their antiviral effects against IAV.

## 2. Results and Discussion

### 2.1. Structure Determination of Glaciapyrroles

**Figure 2:**
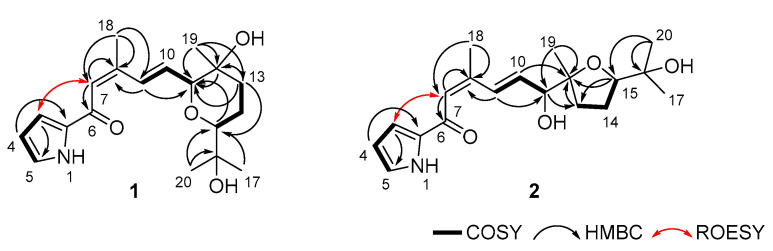
Key COSY, HMBC, and ROESY correlations of glaciapyrroles D and E (**1** and **2**).

Glaciapyrrole D (**1**) was obtained as a white powder after purification by systematic fractionation and separation from the extract of *Streptomyces* sp. (#GGS53) culture. The molecular formula of **1** was established as C_19_H_27_NO_4_ based on its positive HRFABMS (*m/z*: [M+H]^+^ calcd for C_19_H_28_NO_4_ 334.2018; observed 334.2020), suggesting seven degrees of unsaturation. The ^13^C NMR spectrum of **1** exhibited a carbonyl carbon (δ_C_ 182.4) and eight olefinic carbons (δ_C_ 150.1, 135.5, 135.4, 130.0, 126.7, 123.1, 117.6, and 111.2), indicating that glaciapyrrole D must have a bicyclic system. Analysis of the ^13^C NMR and multiplicity-edited HSQC spectra of **1** revealed two methine carbons bearing oxygen (δ_C_ 85.5 and 85.0), two oxygen-bound quaternary carbons (δ_C_ 72.8 and 70.8), two aliphatic methylene carbons (δ_C_ 40.0 and 25.0), and four methyl carbons (δ_C_ 26.2, 25.6, 21.3, and 20.7). The ^1^H NMR spectrum acquired in CD_3_OD exhibited four methyl (δ_H_ 2.13, 1.23, 1.23, and 1.10), four aliphatic (δ_H_ 1.87, 1.72, 1.69, and 1.58), two carbinol (δ_H_ 3.87 and 3.25), and six double bond protons (δ_H_ 7.88, 7.06, 7.00, 6.67, 6.37, and 6.24) (Table 1). Although the molecular formula contained 27 protons, the ^1^H NMR spectrum only showed 24 of them; hence, glaciapyrrole D should possess three exchangeable protons. Analysis of the COSY NMR spectrum revealed three partial fragments of **1** (Figure 2). The COSY correlations of the three double bond protons (H-3 (δ_H_ 7.00), H-4 (δ_H_ 6.24), and H-5 (δ_H_ 7.06)) suggested a C-3-C-4-C-5 spin system. The coupling constants between H-3 and H-4 (^3^*J*_HH_ = 3.9 Hz) and between H-4 and H-5 (^3^*J*_HH_ = 2.5 Hz) indicated that this spin system belongs to a five-membered pyrrole ring. Another partial fragment, including H-9 (δ_H_ 7.88, d, ^3^*J*_HH_ = 16.1 Hz), H-10 (δ_H_ 6.37, dd, ^3^*J*_HH_ = 16.1, 4.3 Hz), and H-11 (δ_H_ 3.87, d, ^3^*J*_HH_ = 4.3 Hz) was assigned based on the COSY correlations of H-10 with H-9 and H-11. The large coupling constant (16.1 Hz) established the *E* geometry between H-9 and H-10. The other partial structure, connected from C-13 to C-15, was determined by the continuous COSY correlations from H_2_-13 (δ_H_ 1.87 and 1.69) to H-15 (δ_H_ 3.25) through H_2_-14 (δ_H_ 1.72 and 1.58). The two spin systems of C-9-C-11/C-13-C-15 were assembled using HMBC correlations from H-11 and H_2_-13 to C-12 (δ_C_ 70.8). The two singlet methyl groups of H_3_-17 and H_3_-20 were connected to the same oxygen-bearing quaternary carbon C-16 (δ_C_ 72.8) by their HMBC couplings with C-16. HMBC correlations between H_3_-17 and H_3_-20 with the C-15 (δ_C_ 85.5) indicated the presence of a 2-hydroxy-2-propyl group. Furthermore, long-range HMBC correlations from H-15 to C-11 (δ_C_ 85.0) revealed a tetrahydropyran ring with a terminal isopropyl alcohol functional group (Figure 2). The HMBC correlation from singlet methyl protons H_3_-19 to C-11, 12, and 13 supported the connectivity of C-19 to C-12 (δ_C_ 70.8). HMBC correlations from singlet methyl H_3_-18 (δ_H_ 2.13) to C-7, C-8 (δ_C_ 150.1), and C-9 extended the partial structure to C-7 via C-8. The HMBC from the olefinic proton H-7 to the carbonyl carbon C-6 (δ_C_ 182.4) established an *α*,*β*-unsaturated carbonyl group by connecting C-6 to C-7. Although a long-range HMBC correlation from the methyl protons H_3_-18 to C-6 supported the assembled substructure from C-6 to C-20, no additional HMBC peaks at C-6 were detected from the unconnected pyrrole moiety. We then utilized ROESY correlations between H-7 and H-3 and connected the pyrrole to C-6. Based on the molecular formula, the two alcohols were assigned to oxygen-bearing carbons C-12 and C-16.

**Figure 3:**
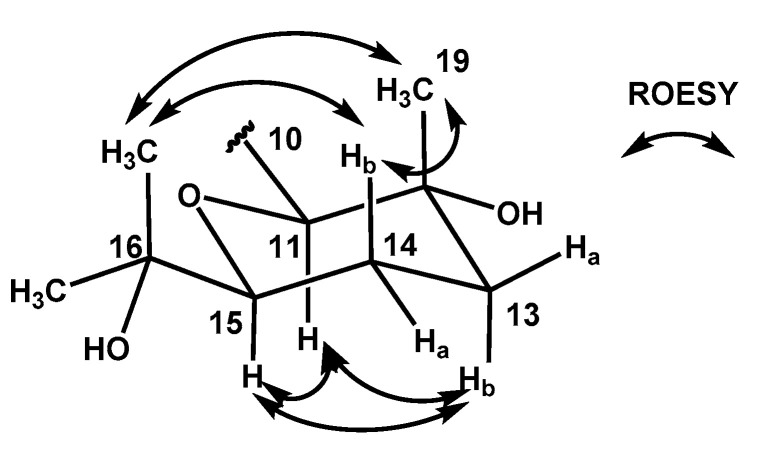
Key ROESY correlations used to determine the relative configuration of tetrahydropyran in **1**.

Precise analysis of the ROESY NMR correlations and ^1^H-^1^H coupling constants of **1** established the relative configuration of the tetrahydropyran moiety. H-15 (δ_H_ 3.25) displayed a doublet of a doublet, and its large coupling constant was measured as 11.4 Hz, which led to the assignment of this proton to an axial position. H-15 was strongly correlated with H-11 (δ_H_ 3.87) and H-13b (δ_H_ 1.69) in the ROESY NMR spectrum, indicating that H-11 and H-13 are also located at axial positions. H_3_-19 (δ_H_ 1.10) displayed a strong ROESY correlation with H-14b (δ_H_ 1.58), leading to their assignments at axial positions on opposite side of H-11 and H-15 (Figure 3). Therefore, the relative configuration of the tetrahydropyran unit was determined as 11S*, 12R*, and 15S*.

Glaciapyrrole D (**1**) does not have any functional group for derivatization with axillary chiral reagents, so as to apply the modified Mosher’s method to determine its absolute configuration. Therefore, electronic circular dichroism (ECD) was used instead for its configurational analysis. The experimental ECD spectrum exhibited the Cotton effects at 220 and 280 nm. Two enantiomers were constructed based on the established relative configurations and then structurally optimized using density functional theory (DFT) modeling for ECD calculations. The calculated ECD spectra of the two enantiomers support 11*S*, 12*R*, and 15*S* configuration for **1** (Figure S41).

Glaciapyrrole E (**2**) was purified as a white powder. Its molecular formula was deduced to be C_19_H_27_NO_4_ from the positive mode HRFABMS (*m/z* [M+H]^+^ calcd for C_19_H_28_NO_4_ 334.2018 found 334.2021). The ^1^H and ^13^C NMR spectra of **2** were similar to those of **1**, displaying the signals for the pyrrole conjugated with dienone and the isopropyl alcohol moiety. However, the ^1^H and ^13^C NMR resonances corresponding to the tetrahydropyran moiety in **1** were noticeably changed in the NMR spectra of **2** (Table 1). Further detailed analysis of the 2D NMR data revealed the modification of the ring structure in **2**. H_2_-14 was correlated with H_2_-13 and H-15 in COSY NMR data, establishing the C-13-C-14-C-15 connectivity. H_3_-19 displayed HMBC correlations with C-11, C-12, and C-13, which led to the assignment of this 3-carbon chain adjacent to C-12 by connecting C-12 and C-13. Next, C-15 was located next to C-16 based on the HMBC correlation to C-15 from the *gem*-dimethyl protons (H_3_-17 and H_3_-20). Finally, a tetrahydrofuran was constructed by the crucial H-15/C-12 HMBC correlation through an ether linkage, elucidating the planar structure of **2**.

**Figure 4:**
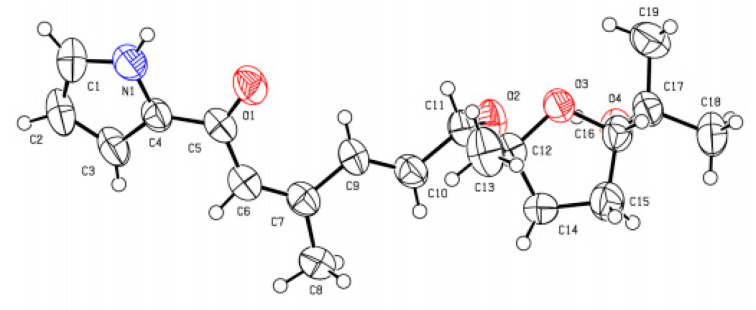
X-ray crystallographic structure of glaciapyrrole E (**2**).

Although the planar structure of **2** was identical to that of previously reported glaciapyrrole A (**3**), **2** eluted much later than **3** in our reversed-phase chromatography analysis. We crystallized **2** in ethyl acetate/hexane to yield a single block type crystal, which diffracted as a monoclinic system for more rigorous structural determination. Single-crystal X-ray diffraction analysis confirmed the planar structure of **2** and determined the relative configuration as 11S*, 12S*, 15R*, whereas **3** possesses an 11S*, 12R*, 15R* configuration. Therefore, **2** (Figure 4) is a 12-epimer of **3** (CCDC Deposition Number 2132067).

**Figure 5:**
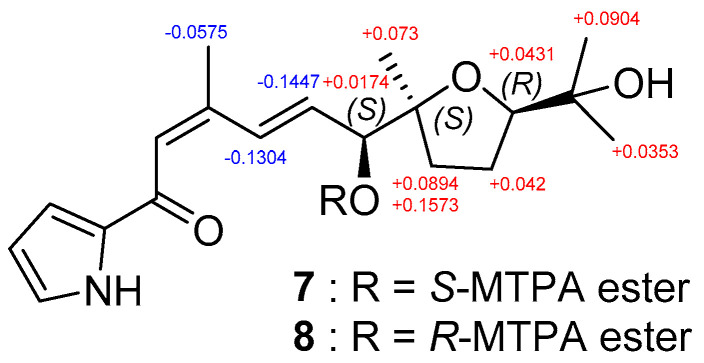
Δδ*_S_*_−_*_R_* values obtained for *S*- and *R*-MTPA esters (**7** and **8**) of glaciapyrrole E (**2**).

The modified Mosher’s method was applied to determine the absolute configuration of **2 [10]**. We derivatized the hydroxy group at C-11 using *R*- and *S*-α-methoxy-α-(trifluoromethyl)phenylacetyl chloride (MTPA-Cl) to produce *S*- and *R*-MTPA esters (**7** and **8**), respectively. Analysis of ^1^H and COSY NMR spectra enabled the assignment of the ^1^H NMR chemical shifts of **7** and **8** and the calculation of their Δδ*_S_*_−_*_R_* values. Based on the Δδ*_S_*_−_*_R_* values, the stereogenic center at C-11 was determined to be *S* (Figure 5). The relative configuration established by X-ray crystallography concurrently assigned the 11*S*, 12*S*, and 15*R* configuration to **2**.

### 2.2. Photoirradiation of Glaciapyrroles

Glaciapyrrole A (**3**) [11] was also obtained from the fermentation of *Streptomyces* sp. GGS53 along with glaciapyrroles D and E (**1**–**2**). During the experiment in a laboratory environment, the photoactivity of the glaciapyrroles was recognized, and a series of experiments were performed to confirm this observation. First, we checked the photoreactivity of the glaciapyrroles by using a post column photolytic reactor with a 366 nm (8 W) UV source on the front line of the LC/MS column. After confirming the photoreactivity of the glaciapyrroles with LC/MS, an optimization process was performed to establish effective photoreaction conditions in a preparative scale. To optimize the photoirradiation conditions, experiments were conducted with various substrate concentrations, irradiation durations, and UV wavelengths. Glaciapyrroles A, D, and E were dissolved in EtOH at 10, 5, or 1 mM, and these solutions were irradiated under 254 or 366 nm UV light at an output power of 8 W for 0, 10, 20, 30, or 40 min. During the optimization processes, the glaciapyrroles were nonspecifically degraded under 254 nm UV radiation, whereas they were systematically converted to specific products under 366 nm. The photoirradiation reactions were monitored by LC/MS. The highest conversion ratios (approximately 50%) were observed under the conditions of 1 mM, 366 nm, and 40 min.

**Figure 6:**
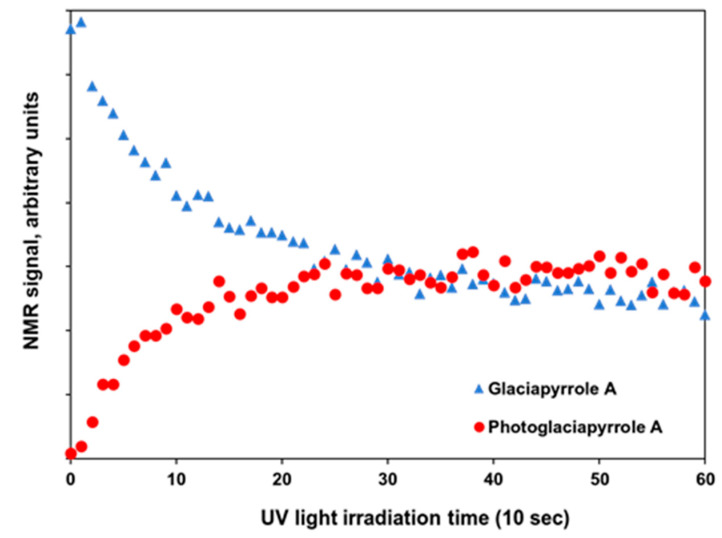
^1^H NMR signal intensity plot of glaciapyrrole A and photoglaciapyrrole A according to UV (366 nm) irradiation time. Each point is an integration value (an arbitrary unit) of ^1^H NMR signals at H-9 position of corresponding compounds.

Time-resolved photoisomerization of the glaciapyrroles was monitored in situ by LED-illuminated NMR spectroscopy [12]. Glaciapyrrole A (10 mM) was dissolved in 400 μL of CD_3_OD. The solution was transferred to an amberized NMR tube, and a coaxial tube of quartz glass was inserted into the tube. Then, an optical fiber coupled with the UV-LED illuminator was inserted into a coaxial tube to guide 366 nm light from the UV-LED illuminator into the sample in the NMR spectrometer. A series of ^1^H NMR spectra were recorded under UV irradiation every 10 sec to monitor the photoisomerization progress. Figure 6 plots the integrated intensity of each H-9 signal of photoglaciapyrrole A and glaciapyrrole A according to the UV irradiation time. The exponential decrease in glaciapyrrole A under UV irradiation implies first-order photochemical reaction kinetics. After 200 sec of UV irradiation, the reactant and product reached an equilibrium state (Figure 6). The equilibrium state was also observed when purified photoglaciapyrrole A (**6**) was photoisomerized reversely to glaciapyrrole A (**3**) by UV 366 nm (Figure S42), indicating that glaciapyrrole A and photoglaciapyrrole A undergo systematic reversible photoisomerization (Figure S43). Subsequently, glaciapyrroles D and E (**1**–**2**) were systematically converted to photoglaciapyrroles D and E (**4**–**5**) under the optimized conditions, respectively.

### 2.3. Structure Determination of Photoglaciapyrroles

**Figure 7:**
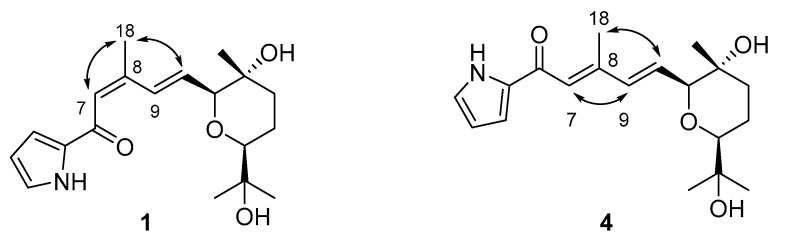
Key ROESY correlations to determine double-bond geometries of glaciapyrrole D (**1**) and photoglaciapyrrole D (**4**).

Photoglaciapyrrole D (**4**) was purified from the photoreaction mixture of **1** as a white powder, and its molecular formula was determined to be C_19_H_27_NO_4_ using HRFABMS (*m/z*: [M+H]^+^ calcd for C_19_H_28_NO_4_ 334.2018; found 334.2022). Interpretation of the ^1^H, ^13^C, COSY, HSQC, and HMBC NMR data indicated that the gross structure of **4** was identical to that of **1** (Table 2). However, a detailed analysis of the ^13^C NMR chemical shifts revealed that C-9 (δ_C_ 135.6) and C-18 (δ_C_ 14.5) displayed distinctively different chemical shifts in **4** compared to those carbons (δ_C_ 130.0 and 21.3) in **1**, indicating a possible geometric change around these carbons. Isomerization of the double-bond geometry was confirmed by ROESY correlations. Glaciapyrrole D (**1**) with 7*Z* configuration displayed H-7/H_3_-18 ROESY correlation, whereas photoglaciapyrrole D (**4**) exhibited an H-7/H-9 ROESY cross peak that led to the assignment of a 7*E* configuration (Figure 7).

Photoglaciapyrrole E (**5**) was isolated as a product of the photoreaction of **2**. Its molecular formula was deduced to be C_19_H_27_NO_4_ using HRFABMS (*m/z*: [M+H]^+^ calcd for C_19_H_28_NO_4_ 334.2018; observed 334.2021). The double-bond geometry of **5** was also established as 7*E* and 9*E* by analyzing the ^13^C and ROESY NMR spectra. Photoglaciapyrrole A (**6**) was obtained as a white powder by photoirradiation of **3**. Analysis of ^13^C and ROESY NMR data revealed the double-bond geometries of photoglaciapyrrole A as 7*E* and 9*E*, which are identical to those of photoglaciapyrroles D and E (**4** and **5**).

### 2.4. Evaluation of Antiviral Activity with Encapsulated Poly(lactic-co-glycolic acid) Nanoparticles

Influenza viruses are classified into types A, B, C, and D based on their nucleic acid and protein composition. Influenza A virus (IAV) has a wide range of hosts and lacks the proofreading function in its RNA polymerase; hence, research on IAV has been gaining importance [13,14,15]. The H1N1, H2N2, and H3N2 subtypes of IAV were responsible for many human cases in previous global influenza pandemics [16]. IAV infects people of all ages through the respiratory pathway, causing fever, headaches and chills, which in severe cases can lead to complications [17]. The negative-sense, single-stranded RNA genome of IAV contains approximately 13,500 bases. This genome is divided into eight segments and encodes at least 17 proteins, including envelope glycol protein (HA), neuraminidase (NA), three polymerases (PA, PB1, and PB2), nucleoprotein (NP), matrix protein (M1), ion channel protein (M2), nonstructural proteins (NS1 and NS2), as well as the recently discovered NS3, M42, PA-N182, and PA-N155 [13,18]. Many antiviral drugs have been developed to target these viral proteins, the viral RNA, or host factors involved in virus replication [14]. However, IAV has not been fully controlled and continues to cause significant disease burden [19]. In our initial screening of **1**–**6** for antiviral activity, glaciapyrrole E (**2**) and photoglaciapyrrole E (**5**) displayed antiviral effects, whereas the other four congeners were not active.

In addition to significant bioactivity, drugs should also be delivered using efficient methods for clinical applications. Poly(lactic-co-glycolic acid) (PLGA) is a polymer approved by the US FDA and EMA for safe administration. Recently, it has received significant attention in biomedical applications owing to its excellent biocompatibility and biodegradability. In particular, PLGA-based nanoparticles (PLGA-NPs) have many advantages in drug delivery systems, such as the controlled and sustained release of entrapped drugs, facile surface modification, ease for long-term storage [20,21,22]. Considering the unique characteristics of PLGA NPs, we fabricated PLGA NPs containing glaciapyrroles or photoglaciapyrroles (Gla-PLGA NPs) to investigate their bioactivities.

To effectively evaluate the antiviral effects of glaciapyrrole E (**2**) and photoglaciapyrrole E (**5**), Gla-PLGA NPs were formulated on a NanoAssemblr using a microfluidic mixing approach. Encapsulation was achieved by dissolving glaciapyrrole E and photoglaciapyrrole E in the solvent phase together with the PLGA polymer and formulating NPs by mixing it with poly(vinyl alcohol) (PVA) as a stabilizer in the aqueous phase. Because the aqueous and solvent phases were rapidly and precisely mixed in this process, Gla-PLGA NPs were precipitated in a controlled manner at the desired size with a narrow size distribution. As shown in Table 3, the hydrodynamic diameters of PLGA NPs (blank), GlaE-PLGA NPs (glaciapyrrole E), and pGlaE-PLGA NPs (photoglaciapyrrole E) were 72.4, 96.3, and 108.7 nm, respectively. The encapsulation efficiencies of glaciapyrrole E and photoglaciapyrrole E in the NPs were found to be 14.4% and 24.6%, respectively. This increase in particle size after encapsulation was potentially due to the adsorption of glaciapyrrole E and photoglaciapyrrole E on the porous surface of PLGA NPs.

**Figure 8:**
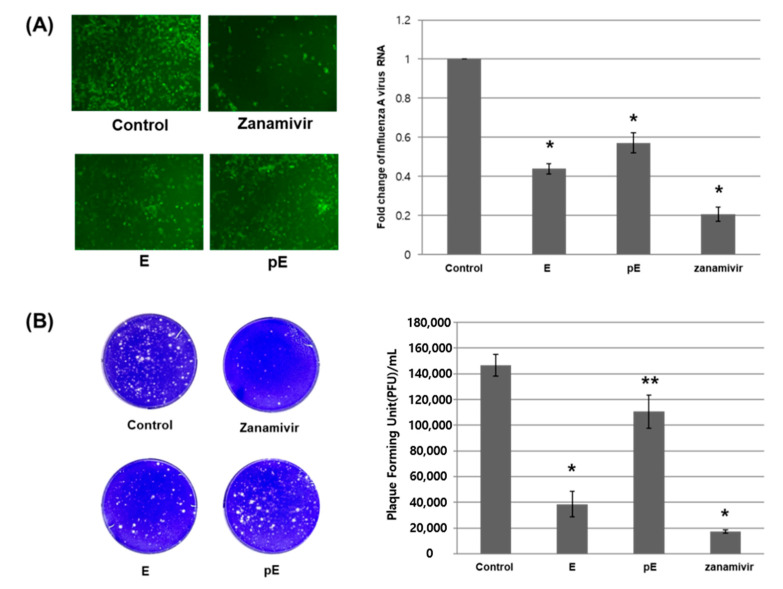
Glaciapyrrole E and photoglaciapyrrole E inhibit the replication of the influenza A virus. MDCK cells were inoculated with IAV and incubated at 37 °C for 1 h. Cells were treated with either PLGA NPs (control), GlaE-PLGA NPs (E), or pGlaE-PLGA NPs (pE), or zanamivir. (**A**) After 24 h, fluorescence images were taken using a fluorescence microscope, and the relative amounts of IAV RNA in cells were determined via qRT-PCR using primer for IAV M1. (**B**) After 48 h, the supernatant was infected with MDCK cells before the plaque assay. The value is the average of triplicate. *p*-values were determined by *t*-test (* *p* < 0.005, ** *p* < 0.05).

Mardin–Darby canine kidney (MDCK) cells were inoculated with IAV at a multiplicity of infection (MOI) of 0.005, followed by treatment with 200 μM of GlaE-PLGA NPs, 200 μM of pGlaE-PLGA NPs, 1 μM of zanamivir (an anti-influenza virus drug as the positive control), or empty particles (negative control). After 24 h of inoculation, green fluorescent protein (GFP) images were captured using fluorescence, and the amount of IAV RNA in cells was estimated using qRT-PCR. In cells treated with the GlaE-PLGA NPs (E) and pGlaE-PLGA NPs (pE), the amount of HEV RNA was about 50% lower than that in the control treatment group (Figure 8A). To ascertain the inhibitory effects of glaciapyrrole E and photoglaciapyrrole E, we re-infected the supernatant at 48 h after inoculation. Based on results from the plaque reduction assay (Figure 8B), photoglaciapyrrole E decreased the viral titer by 25% compared to the control treatment group, whereas glaciapyrrole E reduced the viral titer by more than 70%.

## 3. Materials and Methods

### 3.1. General Experimental Procedures

Optical rotation values were measured at 25 °C using a JASCO P-2000 polarimeter (Tokyo, Japan) with a 1 cm cell. UV and IR spectra were obtained using a Perkin-Elmer Lambda 35 spectrophotometer (Waltham, MA, USA) and a JASCO FT/IR-4200 spectrometer (Tokyo, Japan), respectively. Circular dichroism (CD) spectra were acquired on an Applied Photophysics Chirascan V100 CD spectrometer (Leatherhead, UK) using a 1 mm CD cell. NMR spectra were collected using Bruker Avance 600 and 850 MHz NMR spectrometers (Billerica, MA, USA) located at the National Center for Inter-University Research Facilities (NCIRF), Seoul National University. Low-resolution electrospray ionization (ESI) data were acquired using an Agilent Technologies 6130 quadrupole mass spectrometer (Santa Clara, CA, USA) coupled with an Agilent Technologies 1200-series HPLC with a reversed-phase C_18_ column (Phenomenex Luna, 100 × 4.6 mm). High-resolution FAB mass spectrometric data were obtained using a JEOL JMS 700 mass spectrometer (Tokyo, Japan) at the National Center for Inter-University Research Facilities (NCIRF). HPLC isolation was performed on a Gilson 321 (Middleton, WI, USA) equipped with a UV detector Gilson UV-Vis-151 (Middleton, WI, USA). X-ray crystallographics were collected on a Rigaku R-AXIS RAPID diffractometer (Tokyo, Japan) using graphite-monochromated Mo Kα radiation (λ = 0.71075 Å).

### 3.2. Bacterial Isolation

A deep-sea sediment core (Station 1; ca. 60 cm long) was collected with a multi-corer in the East Sea of Korea (37°12.0486′, 130°25.0889′, ca. 2163 m in water depth) during the RV Araon test cruise in June 2014. The sediment sample (1 g) was diluted in 12 mL of sterilized solar salt water (1:3 dilution), vortexed, and spread onto one of the following actinomycete isolation agar: A4 medium (1 L of seawater, 18 g of agar, and 100 mg/L of cycloheximide), A5 medium (750 mL of seawater, 250 mL of distilled H_2_O, 18 g of agar, and 100 mg/L of cycloheximide), A6 medium (1 L of seawater, 18 g of agar, and 5 mg/L of polymyxin B sulfate), A7 medium (1 L of seawater, 18 g of agar, and 5 mg/L of kanamycin), and chitin-based agar. GGS53 was isolated on the A7 agar medium. The strain culture was repeatedly applied to the solid A1 medium to obtain a single strain. The 16S rDNA sequencing analysis data (GenBank accession number: OK037580) obtained from COSMO Co., Ltd., (Seoul, Korea), revealed that GGS53 is most closely related to *Streptomyces niveus* (99% identity), identifying the strain as a *Streptomyces* sp.

### 3.3. Cultivation and Extraction

The GGS53 strain was cultured in 30 mL of A1 medium (10 g of starch, 4 g of peptone, 1 g of calcium carbonate, and 2 g of yeast extract in 1 L of solar salt water) in a 125-mL Erlenmeyer flask. After 5 days of fermentation on a rotary shaker at 170 rpm at 28 °C, 10 mL of the culture was inoculated in 1 L of A1 medium in 2.8 L Fernbach flasks (48 × 1 L for a total volume of 48 L). The large culture was incubated at 170 rpm and 28 °C. After five days, the whole culture was extracted with EtOAc. The EtOAc layer was separated and dried over anhydrous sodium sulfate. The entire extraction process was repeated twice. The EtOAc extract was enriched in vacuo to yield 3.2 g of dried material.

### 3.4. Isolation of Glaciapyrrole D, E, and A (***1**–**3***)

Celite-adsorbed GGS53 extract was loaded on a 5 g Sep-Pak C_18_ cartridge and fractionated using a step elution with combinations of H_2_O and MeOH (20, 40, 60, 80, and 100% aqueous MeOH). The glaciapyrroles were detected in the 60% MeOH fraction. To isolate the glaciapyrroles, the 60% fraction was chromatographed by semipreparative reversed-phase HPLC (YMC-ODS column, 10 × 250 mm, flow rate 3.3 mL/min, UV 330 nm detection) under isocratic 40% aqueous CH_3_CN condition. Glaciapyrroles D, E, and A were eluted at 13, 21, and 16 min, respectively. Glaciapyrrole E was further purified using semipreparative reversed-phase HPLC (YMC-ODS column, 10 × 250 mm, flow rate 3.3 mL/min, UV 330 nm detection) using an isocratic 63% aqueous MeOH condition (retention time: 18 min). Glaciapyrrole D (**1**) (10 mg), glaciapyrrole E (**2**) (8 mg), and glaciapyrrole A (**3**) (15 mg) were obtained as pure compounds.

Glaciapyrrole D (**1**). White powder; ${\left[\alpha \right]}_{D}^{\mathrm{25}}$ +14 (c 0.1, MeOH); UV (MeOH) λ_max_ (log ε) 207 (3.99) nm, (log ε) 334 (4.13) nm; IR (neat) ν_max_ 3395, 3255, 2974, 1633, 1615, 1573, 1449, 1406, 1312, 1115 cm^−1^; ^1^H and ^13^C NMR data, see Table 1; HRFABMS *m/z* 334.2020 [M+H]^+^ (calculated for C_19_H_28_NO_4_, 334.2018).

Glaciapyrrole E (**2**). White powder; ${\left[\alpha \right]}_{D}^{\mathrm{25}}$ −40 (c 0.1, MeOH); UV (MeOH) λ_max_ (log ε) 204 (3.91) nm, (log ε) 333 (4.12) nm; IR (neat) ν_max_ 3343, 3273, 2972, 1633, 1615, 1572, 1448, 1406, 1315, 1115 cm^−1^; ^1^H and ^13^C NMR data, see Table 1; HRFABMS *m/z* 334.2021 [M+H]^+^ (calculated for C_19_H_28_NO_4_, 334.2018).

### 3.5. X-ray Crystallographic Data of glaciapyrrole E (***2***)

Colorless block crystal, C_19_H_27_NO_4_, *M*_r_ = 333.41, monoclinic, space group C 2 (#5), a = 22.435(5) Å, b = 6.2694(12) Å, c = 15.341(3) Å, α = 90°, β = 118.86(3)°, γ = 90°, V = 1889.8(8) Å^3^, T = 23.0 °C, 2θ_max_ = 2.47, Z = 4, crystal dimensions = 0.200 × 0.110 × 0.110 mm, F_000_ = 720.00, The final R_1_ value 0.0511 (*w*R_2_ = 0.1702) for 3334 all reflections (*I* > 2.00σ(*I*)).

### 3.6. Photoirradiation

Photoirradiation of glaciapyrroles was performed using a MicroSolv Technology Corp (Leland, NC, USA) post column photolytic reactor at 366 nm (8 W). The EtOH solution of the glaciapyrroles (30 mL, 1 mM) was placed in a petri dish (90 × 15 mm), kept 35 mm away from the lamp, and irradiated for 40 min. The EtOH solution was analyzed every 10 min using LC/MS. After 40 min of photoirradiation, the ratio of glaciapyrrole to photoglaciapyrrole was found to stabilize at 1:1. Glaciapyrrole A (**3**) and photoglaciapyrrole A (**6**) were isolated by semi-preparative reversed-phase HPLC (YMC-ODS column, 10 × 250 mm, flow rate 3.3 mL/min, UV 330 nm detection) using an isocratic 36% aqueous CH_3_CN condition. Glaciapyrrole A and photoglaciapyrrole A were eluted at 23 and 15 min, respectively. Glaciapyrrole D (**1**), photoglaciapyrrole D (**4**), glaciapyrrole E (**2**), and photoglaciapyrrole E (**5**) were also purified by HPLC (isocratic 33% aqueous CH_3_CN, *t*_R_ = 24 and 18 min for **1** and **4**; isocratic 38% aqueous CH_3_CN, *t*_R_ = 22 and 16 min for **2** and **5**, respectively).

Photoglaciapyrrole D (**4**). White powder; ${\left[\alpha \right]}_{D}^{\mathrm{25}}$ +17 (c 0.1, MeOH); UV (MeOH) λ_max_ (log ε) 204 (3.76) nm, (log ε) 333 (4.04) nm; IR (neat) ν_max_ 3403, 3276, 2975, 1618, 1578, 1542, 1409, 1385, 1313, 1113 cm^−1^; ^1^H and ^13^C NMR data, see Table 1; HRFABMS *m/z* 334.2022 [M+H]^+^ (calculated for C_19_H_28_NO_4_, 334.2018).

Photoglaciapyrrole E (**5**). White powder; ${\left[\alpha \right]}_{D}^{\mathrm{25}}$ −21 (c 0.1, MeOH); UV (MeOH) λ_max_ (log ε) 201 (4.13) nm, (log ε) 332 (4.27) nm; IR (neat) ν_max_ 3388, 3274, 2971, 1618, 1577, 1407, 1313, 1111 cm^−1^; ^1^H and ^13^C NMR data, see Table 1; HRFABMS *m/z* 334.2021 [M+H]^+^ (calculated for C_19_H_28_NO_4_, 334.2018).

Photoglaciapyrrole A (**6**). White powder; ${\left[\alpha \right]}_{D}^{\mathrm{25}}$ +12 (c 0.1, MeOH); UV (MeOH) λ_max_ (log ε) 203 (3.81) nm, (log ε) 333 (4.10) nm; IR (neat) ν_max_ 3417, 3272, 2973, 1619, 1579, 1408, 1313, 1113 cm^−1^; ^1^H and ^13^C NMR data, see Table 1; HRFABMS *m/z* 334.2017 [M+H]^+^ (calculated for C_19_H_28_NO_4_, 334.2018).

### 3.7. MTPA Esterification of Glaciapyrrole E

Glaciapyrrole E (**2**, 1.5 mg) was transferred to each of two 20 mL vials that had been completely dried under high vacuum for 5 h. First, Ar gas was injected into each vial, and 1 mL of completely distilled pyridine was added. The reaction mixture was then stirred at room temperature for 5 min. After 5 min, R- and S-α-methoxy-α-(trifluoromethyl)phenylacetyl chloride (MTPA-Cl) (25 μL) were separately added. The reactions were terminated after 15 min by adding 50 μL of MeOH. The mixtures were then injected into a reversed-phase HPLC (YMC-ODS column, 10 × 250 mm, flow rate 2 mL/min, UV 330 nm detection). The products were purified under a step gradient solvent system (50–90% aqueous CH_3_CN for 30 min, 90% aqueous CH_3_CN from 30 to 60 min, and 100% CH_3_CN from 60 to 80 min). The S-MTPA ester (**7**) and R-MTPA ester (**8**) eluted at 32.3 and 33 min, respectively. The molecular formula of the two products were determined to be C_29_H_35_F_3_NO_6_ by ESI-MS ([M+H]^+^ *m/z* at 549). The Δδ*_S_*_−_*_R_* values of the S- and R-MTPA esters were calculated based on analyzing their ^1^H and COSY NMR spectral data.

S-MTPA ester of glaciapyrrole D (**7**). White powder; ^1^H NMR (500 MHz, CD_3_OD-d_4_) δ 7.80 (1H, d, *J* = 16.2 Hz), 7.49–7.45 (2H, m), 7.37–7.30 (3H, m), 7.08 (1H, dd, *J* = 2.4, 1.3 Hz), 6.99 (1H, dd, *J* = 3.8, 1.3 Hz), 6.71 (1H, s), 6.25 (1H, dd, *J* = 3.8, 2.4 Hz), 6.17 (1H, dd, *J* = 16.2, 6.8 Hz), 5.55 (1H, d, *J* = 6.8 Hz), 3.84 (1H, dd, *J* = 8.4, 6.5 Hz), 3.56 (3H, s), 2.06 (3H, s), 2.01–1.83 (3H, m), 1.77 (1H, m),1.24 (3H, s), 1.19 (3H, s), 1.15 (3H, s)

R-MTPA ester of glaciapyrrole D (**8**). White powder; ^1^H NMR (500 MHz, CD_3_OD-d_4_) δ 7.93 (1H, d, *J* = 16.2 Hz), 7.56–7.52 (2H, m), 7.47–7.38 (3H, m), 7.08 (1H, dd, *J* = 2.4, 1.3 Hz), 7.01 (1H, dd, *J* = 3.8, 1.3 Hz), 6.74 (1H, s), 6.32 (1H, dd, *J* = 16.2, 6.7 Hz), 6.25 (1H, dd, *J* = 3.8, 2.4 Hz), 5.53 (1H, d, *J* = 6.8 Hz), 3.80, (1H, dd, *J* = 8.4, 6.5 Hz), 3.60 (3H, s), 2.12 (3H, s), 1.90–1.18 (3H, m), 1.60 (1H, m), 1.15 (3H, s), 1.12 (3H, s), 1.11 (3H, s)

### 3.8. ECD Calculation

Possible enantiomers of **1** were generated based on ROESY NMR data using Avogadro 1.2.0, and their geometries were optimized in the ground-state based on density functional theory (DFT) calculation using Turbomole X 4.3.2. The B3LYP/DFT level and def-SV(P) basis set were used for the calculations. The ECD spectra were simulated by overlapping each transition, where *σ* is the width of the band at the height of 1/*e*. Δ*Ei* is the excitation energy, and *Ri* is the rotatory strength for transition *i*. In this calculation, the *σ* value was 0.10 eV.

$$\Delta \epsilon \left(E\right)=\frac{1}{2.297\times 10^{-39}}\frac{1}{\sqrt{2\pi \sigma }}\overset{A}{\underset{i}{\sum }}\Delta E_{i}R_{i}e^{[-{\left(E-\Delta E_{i}\right)}^{2}/\left(2\sigma {)}^{2}\right]}$$

### 3.9. Synthesis and Characterization of Gla-PLGA NPs

Gla-PLGA NPs were produced using the NanoAssemblr Platform (Precision NanoSystems Inc., Vancouver, BC, Canada) in a Y-shaped staggered herringbone micromixer with a width of 300 μm and a height of 130 μm. The NPs were synthesized by dissolving 2 mg of PLGA and 2 mg of GlaE (or pGlaE) in 1 mL of CH_3_CN. The resulting mixture was injected into one inlet port on the NanoAssemblr Platform. Simultaneously, 1 mL of PVA solution (2% *w*/*v*) was injected into the other inlet port of the platform. Under a flow rate ratio of 1:1 and a total flow rate of 8 mg/min condition, Gla-PLGA NPs were fabricated using the NanoAssemblr Platform. The produced samples were dialyzed against 1 L PBS using a dialysis bag (MWCO-10 kDa) for 12 h, replacing the dialysis medium twice in the first 4 h, followed by lyophilization with 2% trehalose. The size of Gla-PLGA NPs was measured using a particle size analyzer (Nano ZS90, Malvern Instruments, Worcs, UK). The encapsulation efficiency of Gla into PLGA NPs was determined using LC/MS quantitative analysis after the capsulation disruption with solvent. Gla-PLGA NPs were dissolved in MeOH, and LC/MS analysis was performed with a column Phenomenex C_18_ (4.6 × 150 mm, flow rate 0.7 mL/min, UV 310 nm detection). The mobile phases were MeOH and H_2_O with 10 mM ammonium formate, and the analytical condition was a linear gradient from 60% aqueous MeOH to 100% MeOH in 20 min. All samples were analyzed based on a standard curve of free GlaE and pGlaE, respectively. The following equation was used to calculate the encapsulation efficiency of Gla-PLGA NPs.

Encapsulation efficiency (%) = (weight of the drug in nanoparticles/weight of the feeding drugs) × 100

### 3.10. Cells and Viruses

Mardin–Darby canine kidney(MDCK) cells, and influenza A virus PR8-GFP were provided by Prof. Man-Seong Park (Korea University). MDCK cells were cultured in Minimum Essential Medium (MEM; Hyclone) supplemented with 10% FBS and 1% P/S.

### 3.11. Quantitative RT-PCR (qRT-PCR)

Quantitative analysis of IAV RNA in cells was isolated using a total RNA prep kit (Biofact, Daejeon Korea) and reverse-transcribed to cDNA using a TOPscript™ cDNA Synthesis kit (Enzynomics, Daejeon, Korea). Real-time PCR was performed using the 1X HOT FIREPol EvaGreen qPCR Mix Plus (Solis BioDyne, Tartu, Estonia). The primer sequences used for amplification were as follows: IAV M1, 5′-GACCAATCCTGTCACCTCTGAC-3′ (forward) and 5′-AGGGCATTTTGGACAAACCGTCTA-3′ (reverse); and Canis lupus familiaris glyceraldehyde-3-phosphate dehydrogenase (GAPDH), 5′-CCAGGGCTGCTTTTAACTCTGG-3′ (forward) and 5′-ACTGTGCCGTGGAATTTGCCG-3′ (reverse).

### 3.12. Plaque Assay

MDCK cells were infected with IAV and treated with Gla (200 µM) or zanamivir (1 µM). After 48 h of infection, cell supernatants were collected for virus titration. They were then inoculated with serial diluted supernatant and incubated at 37 °C for an hour, then a mixture of 4% agarose was added and 2× DMEM 0.4% BSA, 25 mM HEPES, and 1% P/S was added. After 2 days, 10% formaldehyde was treated for 2 h and then dyed with 0.3% crystal violet to observe.

### 3.13. Cell Viability Assay

Cell viability was analyzed using MTT (Sigma, Ronkonkoma, NY, USA), as described previously [23].

### 3.14. In-Situ LED-Illuminated NMR Spectroscopy

In-situ LED-illuminated NMR spectra were recorded using a Bruker Avance-III 400 MHz NMR spectrometer (Billerica, MA, USA) equipped with a BBO probe. UV light (366 nm) was generated from a five-channel UV-LED illuminator (Prizmatix, Holon, Israel). An optic fiber (1 m length and 1000 μm core size), whose distal part corresponds to the NMR detection range was sandblasted for uniform light irradiation [24]. A single pulse experiment (zg30) was employed for NMR spectrum acquisition. A series of ^1^H spectra were acquired at intervals of 10 s under continuous UV light irradiation. Each ^1^H spectrum was obtained for 4 s with one scan number.

## 4. Conclusions

Two new pyrrolosesquiterpenes, glaciapyrrole D (**1**) and glaciapyrrole E (**2**), were discovered from *Streptomyces* sp. in deep-sea sediment. The structures of **1** and **2** were determined by the analyzing the combined spectroscopic data, crystallographic data, chiroptical chemical derivatization, and ECD calculation results. **1** bears a tetrahydropyran moiety, whereas **2** contains a tetrahydrofuran substructure derived from alternative cyclization of the sesquiterpenoid chains. The tetrahydropyran structure has not been reported for the glaciapyrrole class before, indicating its structural uniqueness. Furthermore, we designed the experiments for systematic conversion of the glaciapyrroles to the corresponding photoglaciapyrroles by UV photoirradiation. Applying this photoirradiation method, the photoreactivity of the glaciapyrroles was identified, and the photoinduced double-bond isomerization led to structural diversification of these pyrrole-bearing sesquiterpenoids. The NanoAssemblr platform was used to efficiently investigate the biological activity of the glaciapyrrole family compounds, which have only been subjected to limited studies before. Specifically, the generation of encapsulated PLGA nanoparticles allowed us to evaluate their antiviral effects for the first time. Glaciapyrrole E and photoglaciapyrrole E displayed inhibitory activity against influenza A virus, with a higher activity with glaciapyrrole E. These results highlight the potential of combining the study of actinobacteria in deep-sea habitats with photochemical structural diversification for utilizing bioactive molecules.

## Figures and Tables

**Table 1:** NMR data of glaciapyrroles D-E (**1**-**2**) in methanol-*d*_4_.

Position	1 *^a^*			2 *^a^*		
	δ_C_	Type	δ_H_, mult (*J* in Hz)	δ_C_	Type	δ_H_, mult (*J* in Hz)
1			NH			NH
2	135.5	C		135.3	C	
3	117.6	CH	7.00, dd (3.9, 1.4)	117.4	CH	6.98, dd (3.9, 1.3)
4	111.2	CH	6.24, dd (3.9, 2.5)	111.2	CH	6.23, dd (3.9, 2.5)
5	126.7	CH	7.06, dd (2.5, 1.4)	126.6	CH	7.06, dd (2.5, 1.3)
6	182.4	C		182.1	C	
7	123.1	CH	6.67, s	123.6	CH	6.68, s
8	150.1	C		149.6	C	
9	130.0	CH	7.88, d (16.1, 1.3)	131.5	CH	7.86, d (16.0)
10	135.4	CH	6.37, dd (16.1, 4.3)	137.5	CH	6.21, dd (16.0, 6.8)
11	85.0	CH	3.87, dd (4.3, 1.3)	79.2	CH	4.16, dd (6.8, 1.0)
12	70.8	C		86.8	C	
13a	40.0	CH_2_	1.90–1.86, m	33.6	CH_2_	2.17, ddd (12.4, 8.6, 6.0)
13b			1.71–1.67, m			1.58, dt (12.4, 8.0)
14a	25.0	CH_2_	1.75–1.71, m	27.5	CH_2_	1.96–1.89, m
14b			1.61–1.56, m			
15	85.5	CH	3.25, dd (11.4, 2.2)	86.4	CH	3.84, t (7.3)
16	72.8	C		72.6	C	
17	25.6	CH_3_	1.23, s	27.3	CH_3_	1.12, s
18	21.3	CH_3_	2.13, s	21.3	CH_3_	2.10, s
19	20.7	CH_3_	1.10, s	24.1	CH_3_	1.17, s
20	26.2	CH_3_	1.23, s	25.8	CH_3_	1.24, s

*^a^* ^1^H 600 MHz, ^13^C 150 MHz. δ_H_ and δ_C_ values referenced to internal solvent for CD_3_OD at 3.31 ppm and 49.0 ppm.

**Table 2:** NMR data of photoglaciapyrroles D, E and A (**4**–**6**) in methanol-*d*_4_.

Position	4 *^a^*			5 *^a^*			6 *^a^*		
	δ_C_	Type	δ_H_, mult (*J* in Hz)	δ_C_	Type	δ_H_, mult (*J* in Hz)	δ_C_	Type	δ_H_, mult (*J* in Hz)
1			NH			NH			NH
2	135.7	C		135.6	C		135.6	C	
3	117.2	CH	7.01, d (3.8)	117.3	CH	7.01, d (3.8)	117.2	CH	6.99, d (3.7)
4	111.2	CH	6.24, dd (3.8, 2.5)	111.2	CH	6.24, dd (3.8, 2.5)	111.2	CH	6.24, dd (3.7, 2.5)
5	126.5	CH	7.06, d (2.5)	126.6	CH	7.07, d (2.5)	126.5	CH	7.06, d (2.5)
6	183.0	C		182.8	C		182.9	C	
7	124.9	CH	6.79, s	125.3	CH	6.80, s	125.0	CH	6.78, s
8	151.5	C		151.1	C		151.2	C	
9	135.6	CH	6.61, d (15.7)	136.8	CH	6.57, d (15.8)	136.3	CH	6.54, d (16.0)
10	134.3	CH	6.38, dd (15.7, 4.3)	136.6	CH	6.27, dd (15.8, 6.2)	136.9	CH	6.29, dd (16.0, 6.0)
11	85.0	CH	3.87, d (4.3)	78.8	CH	4.21, d (6.2)	78.4	CH	4.16, d (6.0)
12	70.8	C		86.9	C		86.7	C	
13a	40.1	CH_2_	1.90–1.87, m	33.6	CH_2_	2.18–2.14, m	34.7	CH_2_	2.15–2.10, m
13b			1.71–1.67, m			1.61–1.57, m			1.65–1.62, m
14a	25.1	CH_2_	1.75–1.72, m	27.6	CH_2_	1.97–1.91, m (2H)	27.7	CH_2_	1.91–1.87, m
14b			1.61–1.55, m						1.85–1.80, m
15	85.6	CH	3.26, dd (11.3, 1.9)	86.5	CH	3.85, dd (7.3)	88.5	CH	3.85, dd (9.7, 5.9)
16	72.8	C		72.7	C		72.3	C	
17	25.7	CH_3_	1.24, s	25.9	CH_3_	1.13, s	26.3	CH_3_	1.18, s
18	14.5	CH_3_	2.35, s	14.6	CH_3_	2.34, s	14.6	CH_3_	2.33, s
19	20.7	CH_3_	1.11, s	24.1	CH_3_	1.18, s	23.7	CH_3_	1.16, s
20	26.0	CH_3_	1.25, s	27.3	CH_3_	1.25, s	25.1	CH_3_	1.16, s

*^a^* ^1^H 850 MHz, ^13^C 212.5 MHz. δ_H_ and δ_C_ values referenced to internal solvent for CD_3_OD at 3.31 ppm and 49.0 ppm.

**Table 3:** Size properties and encapsulation efficiency of Gla-PLGA NPs.

Sample	Size	Poly Disperse Index (PDI)	Encapsulation Efficiency (%)
GlaE-PLGA NPs	96.3 ± 3.5	0.08 ± 0.03	14.4 ± 2.3
pGlaE-PLGA NPs	108.7 ± 2.5	0.12 ± 0.07	24.6 ± 1.5
PLGA NPs	72.4 ± 5.8	0.12 ± 0.07	-

## Data Availability

Not applicable.

## Supplementary Materials

The following are available online at https://www.mdpi.com/article/10.3390/md20050281/s1, Figure S1. ^1^H NMR spectrum (600 MHz) of glaciapyrrole D (**1**) in MeOH-d_4_, Figure S2. ^13^C NMR spectrum (150 MHz) of glaciapyrrole D (**1**) in MeOH-*d*_4_, Figure S3. COSY spectrum (600 MHz) of glaciapyrrole D (**1**) in MeOH-*d*_4_, Figure S4. ROESY spectrum (600 MHz) of glaciapyrrole D (**1**) in MeOH-*d*_4_, Figure S5. HSQC spectrum (600 MHz) of glaciapyrrole D (**1**) in MeOH-*d*_4_, Figure S6. HMBC spectrum (600 MHz) of glaciapyrrole D (**1**) in MeOH-*d*_4_, Figure S7. ^1^H NMR spectrum (600 MHz) of glaciapyrrole E (**2**) in MeOH-*d*_4_, Figure S8. ^13^C NMR spectrum (150 MHz) of glaciapyrrole E (**2**) in MeOH-*d*_4_, Figure S9. COSY spectrum (600 MHz) of glaciapyrrole E (**2**) in MeOH-*d*_4_, Figure S10. ROESY spectrum (600 MHz) of glaciapyrrole E (**2**) in MeOH-*d*_4_, Figure S11. HSQC spectrum (600 MHz) of glaciapyrrole E (**2**) in MeOH-*d*_4_, Figure S12. HMBC spectrum (600 MHz) of glaciapyrrole E (2) in MeOH-*d*_4_, Figure S13. ^1^H NMR spectrum (850 MHz) of photoglaciapyrrole D (**4**) in MeOH-*d*_4_, Figure S14. ^13^C NMR spectrum (212.5 MHz) of photoglaciapyrrole D (**4**) in MeOH-*d*_4_, Figure S15. COSY spectrum (850 MHz) of photoglaciapyrrole D (**4**) in MeOH-*d*_4_, Figure S16. ROESY spectrum (850 MHz) of photoglaciapyrrole D (**4**) in MeOH-*d*_4_, Figure S17. HSQC spectrum (850 MHz) of photoglaciapyrrole D (**4**) in MeOH-*d*_4_, Figure S18. HMBC spectrum (850 MHz) of photoglaciapyrrole D (**4**) in MeOH-*d*_4_, Figure S19. ^1^H NMR spectrum (850 MHz) of photoglaciapyrrole E (**5**) in MeOH-*d*_4_, Figure S20. ^13^C NMR spectrum (212.5 MHz) of photoglaciapyrrole E (**5**) in MeOH-*d*_4_, Figure S21. COSY spectrum (850 MHz) of photoglaciapyrrole E (**5**) in MeOH-*d*_4_. Figure S22. ROESY spectrum (850 MHz) of photoglaciapyrrole E (**5**) in MeOH-*d*_4_, Figure S23. HSQC spectrum (850 MHz) of photoglaciapyrrole E (5) in MeOH-*d*_4_, Figure S24. HBMC spectrum (850 MHz) of photoglaciapyrrole E (**5**) in MeOH-*d*_4_, Figure S25. ^1^H NMR spectrum (850 MHz) of photoglaciapyrrole A (**6**) in MeOH-*d*_4_, Figure S26. ^13^C NMR spectrum (212.5 MHz) of photoglaciapyrrole A (**6**) in MeOH-*d*_4_, Figure S27. COSY spectrum (850 MHz) of photoglaciapyrrole A (**6**) in MeOH-*d*_4_, Figure S28. ROESY spectrum (850 MHz) of photoglaciapyrrole A (**6**) in MeOH-*d*_4_, Figure S29. HSQC spectrum (850 MHz) of photoglaciapyrrole A (**6**) in MeOH-*d*_4_, Figure S30. HMBC spectrum (850 MHz) of photoglaciapyrrole A (**6**) in MeOH-*d*_4_, Figure S31. ^1^H NMR spectrum (500 MHz) of glaciapyrrole A (**3**) in MeOH-*d*_4_, Figure S32. ^1^H NMR spectrum (500 MHz) of *S*-MTPA ester (**7**) for glaciapyrrole E (**2**) in MeOH-*d*_4_, Figure S33. COSY spectrum (500 MHz) of *S*-MTPA ester (**7**) for glaciapyrrole E (**2**) in MeOH-*d*_4_, Figure S34. ^1^H NMR spectrum (500 MHz) of *R*-MTPA ester (**8**) for glaciapyrrole E (**2**) in MeOH-*d*_4_, Figure S35. COSY spectrum (500 MHz) of *R*-MTPA ester (**8**) for glaciapyrrole E (**2**) in MeOH-*d*_4_, Figure S36. HR-FAB-MS data of glaciapyrrole D (**1**), Figure S37. HR-FAB-MS data of glaciapyrrole E (**2**), Figure S38. HR-FAB-MS data of photoglaciapyrrole D (**4**), Figure S39. HR-FAB-MS data of photoglaciapyrrole E (**5**), Figure S40. HR-FAB-MS data of photoglaciapyrrole A (**6**), Figure S41. Comparison of the experimental and calculated ECD spectra of glaciapyrrole D (**1**), Figure S42. ^1^H NMR signal intensity plot of photoglaciapyrrole A and glaciapyrrole A according to UV (366 nm) irradiation time when purified photoglaciapyrrole was irradiated, Figure S43. A proposed reversible mechanism for the double-bond photoisomerism, Table S1. Cartesian coordinates of glaciapyrrole D (**1**), Table S2. Cartesian coordinates of ent-glaciapyrrole D (**1**), Table S3. ECD calculations of glaciapyrrole D (**1**).
