# Supplementary material for: Discovery and Photoisomerization of New Pyrrolosesquiterpenoids Glaciapyrroles D and E, from Deep-Sea Sediment Streptomyces sp
Source: Mar Drugs. 2022 Apr 22;20(5):281. doi: 10.3390/md20050281 (PMC9147834; doi:10.3390/md20050281)
Supplement: Supplementary file 1 [file marinedrugs-20-00281-s001.zip › marinedrugs-1665545-supplementary.pdf]

Supplementary Materials

Discovery and Photoisomerization of New Pyrrolosesquiterpenoids Glaciapyrroles D and E, from Deep-Sea Sediment *Streptomyces* sp.

Keebeom Ko,[#,1] Seong-Hwan Kim,[#,2] Subin Park,[3] Hwa Seung Han,[2] Jae Kyun Lee,[4] Jin Wook Cha,[2] Sunghoon Hwang,[1] Ki Young Choi,[2] Yoon-Jae Song,[3] Sang-Jip Nam,[5] Seung-Il Nam,[6] Hak Cheol Kwon,[2] Jin-Soo Park*[,2], Dong-Chan Oh*[,1]

[1]Natural Products Research Institute, College of Pharmacy, Seoul National University, 1 Gwanak-ro, Gwanak-gu, Seoul 08826, Republic of Korea

[2]Natural Product Informatics Research Center, Korea Institute of Science and Technology (KIST) Gangneung Institute, Gangneung 25451, Republic of Korea

[3]Department of Life Science, Gachon University, Seongnam-daero 1342 Sujeong-gu, Seongnam, 13120, Republic of Korea

[4]Neuro-Medicine, Korea Institute of Science and Technology, Hwarang-ro 14-gil 5, Seongbuk-gu, Seoul, 02792, Republic of Korea

[5]Department of Chemistry and Nanoscience, Ewha Womans University, Seoul 03760, Republic of Korea

[6]Division of Glacial Environment Research, Korea Polar Research Institute, Incheon 2 1990, Republic of Korea

*J.-S. Park, Tel: +82-33-650-3509, Fax: +82-33-650-3629, E-mail: jinsoopark@kist.re.kr.

*D.-C. Oh, Tel: +82-2-880-2492, Fax: +82-2-762-8322, E-mail: dongchanoh@snu.ac.kr.

Table of Contents

S2-S4 : Table of Contents

S5 : **Figure S1**. ^{1}H NMR spectrum (600 MHz) of glaciapyrrole D (**1**) in MeOH-d_4.

S5 : **Figure S2**. ^{13}C NMR spectrum (150 MHz) of glaciapyrrole D (**1**) in MeOH-d_4.

S6 : **Figure S3**. COSY spectrum (600 MHz) of glaciapyrrole D (**1**) in MeOH-d_4.

S6 : **Figure S4**. ROESY spectrum (600 MHz) of glaciapyrrole D (**1**) in MeOH-d_4.

S7 : **Figure S5**. HSQC spectrum (600 MHz) of glaciapyrrole D (**1**) in MeOH-d_4.

S7 : **Figure S6**. HMBC spectrum (600 MHz) of glaciapyrrole D (**1**) in MeOH-d_4.

S8 : **Figure S7**. ^{1}H NMR spectrum (600 MHz) of glaciapyrrole E (**2**) in MeOH-d_4.

S8 : **Figure S8**. ^{13}C NMR spectrum (150 MHz) of glaciapyrrole E (**2**) in MeOH-d_4.

S9 : **Figure S9**. COSY spectrum (600 MHz) of glaciapyrrole E (**2**) in MeOH-d_4.

S9 : **Figure S10**. ROESY spectrum (600 MHz) of glaciapyrrole E (**2**) in MeOH-d_4.

S10 : **Figure S11**. HSQC spectrum (600 MHz) of glaciapyrrole E (**2**) in MeOH-d_4.

S10 : **Figure S12**. HMBC spectrum (600 MHz) of glaciapyrrole E (**2**) in MeOH-d_4.

S11 : **Figure S13**. ^{1}H NMR spectrum (850 MHz) of photoglaciapyrrole D (**4**) in MeOH-d_4.

S11 : **Figure S14**. ^{13}C NMR spectrum (212.5 MHz) of photoglaciapyrrole D (**4**) in MeOH-d_4.

S12 : **Figure S15**. COSY spectrum (850 MHz) of photoglaciapyrrole D (**4**) in MeOH-d_4.

S12 : **Figure S16**. ROESY spectrum (850 MHz) of photoglaciapyrrole D (**4**) in MeOH-d_4.

S13 : **Figure S17**. HSQC spectrum (850 MHz) of photoglaciapyrrole D (**4**) in MeOH-d_4.

S13 : **Figure S18**. HMBC spectrum (850 MHz) of photoglaciapyrrole D (**4**) in MeOH-d_4.

S14 : **Figure S19**. ^{1}H NMR spectrum (850 MHz) of photoglaciapyrrole E (**5**) in MeOH-d_4.

S14 : **Figure S20**. ^{13}C NMR spectrum (212.5 MHz) of photoglaciapyrrole E (**5**) in MeOH-d_4.

S15 : **Figure S21**. COSY spectrum (850 MHz) of photoglaciapyrrole E (**5**) in MeOH-d_4.

S15 : **Figure S22**. ROESY spectrum (850 MHz) of photoglaciapyrrole E (**5**) in MeOH-d_4.

S16 : **Figure S23**. HSQC spectrum (850 MHz) of photoglaciapyrrole E (**5**) in MeOH-d_4.

S16 : **Figure S24**. HBMC spectrum (850 MHz) of photoglaciapyrrole E (**5**) in MeOH-d_4.

S17 : **Figure S25**. ^{1}H NMR spectrum (850 MHz) of photoglaciapyrrole A (**6**) in MeOH-d_4.

S17 : **Figure S26**. ^{13}C NMR spectrum (212.5 MHz) of photoglaciapyrrole A (**6**) in MeOH-d_4.

S18 : **Figure S27**. COSY spectrum (850 MHz) of photoglaciapyrrole A (**6**) in MeOH-d_4.

S18 : **Figure S28**. ROESY spectrum (850 MHz) of photoglaciapyrrole A (**6**) in MeOH-d_4.

S19 : **Figure S29**. HSQC spectrum (850 MHz) of photoglaciapyrrole A (**6**) in MeOH-d_4.

S19 : **Figure S30**. HMBC spectrum (850 MHz) of photoglaciapyrrole A (**6**) in MeOH-d_4.

S20 : **Figure S31**. ^{1}H NMR spectrum (500 MHz) of glaciapyrrole A (**3**) in MeOH-d_4.

S20: **Figure S32**. ^{1}H NMR spectrum (500 MHz) of *S*-MTPA ester (**7**) for glaciapyrrole E (**2**) in MeOH-d_4.

S21 : **Figure S33**. COSY spectrum (500 MHz) of *S*-MTPA ester (**7**) for glaciapyrrole E (**2**) in MeOH-d_4.

S21 : **Figure S34**. ^{1}H NMR spectrum (500 MHz) of *R*-MTPA ester (**8**) for glaciapyrrole E (**2**) in MeOH-d_4.

S22 : **Figure S35**. COSY spectrum (500 MHz) of *R*-MTPA ester (**8**) for glaciapyrrole E (**2**) in MeOH-d_4.

S22 : **Figure S36**. HR-FAB-MS data of glaciapyrrole D (**1**)

S23 : **Figure S37**. HR-FAB-MS data of glaciapyrrole E (**2**)

S23 : **Figure S38**. HR-FAB-MS data of photoglaciapyrrole D (**4**)

S24 : **Figure S39**. HR-FAB-MS data of photoglaciapyrrole E (**5**)

S24 : **Figure S40**. HR-FAB-MS data of photoglaciapyrrole A (**6**)

S25 : **Figure S41**. Comparison of the experimental and calculated ECD spectra of glaciapyrrole D (**1**).

S25 : **Figure S42**. ^{1}H NMR signal intensity plot of photoglaciapyrrole A and glaciapyrrole A according to UV (366 nm) irradiation time when purified photoglaciapyrrole was irradiated. Each point is an integration value (an arbitrary unit) of ^{1}H NMR signals at H-9 position of corresponding compounds.

S26 : **Figure S43**. A proposed reversible mechanism for the double bond photoisomerism.

S26 : **Table S1**. Cartesian coordinates of glaciapyrrole D (**1**)

S27 : **Table S2**. Cartesian coordinates of *ent*-glaciapyrrole D (**1**)

S28-30 : **Table S3**. ECD calculations of glaciapyrrole D (**1**)

Figure S1. ^{1}H NMR spectrum (600 MHz) of glaciapyrrole D (**1**) in MeOH-d_4.

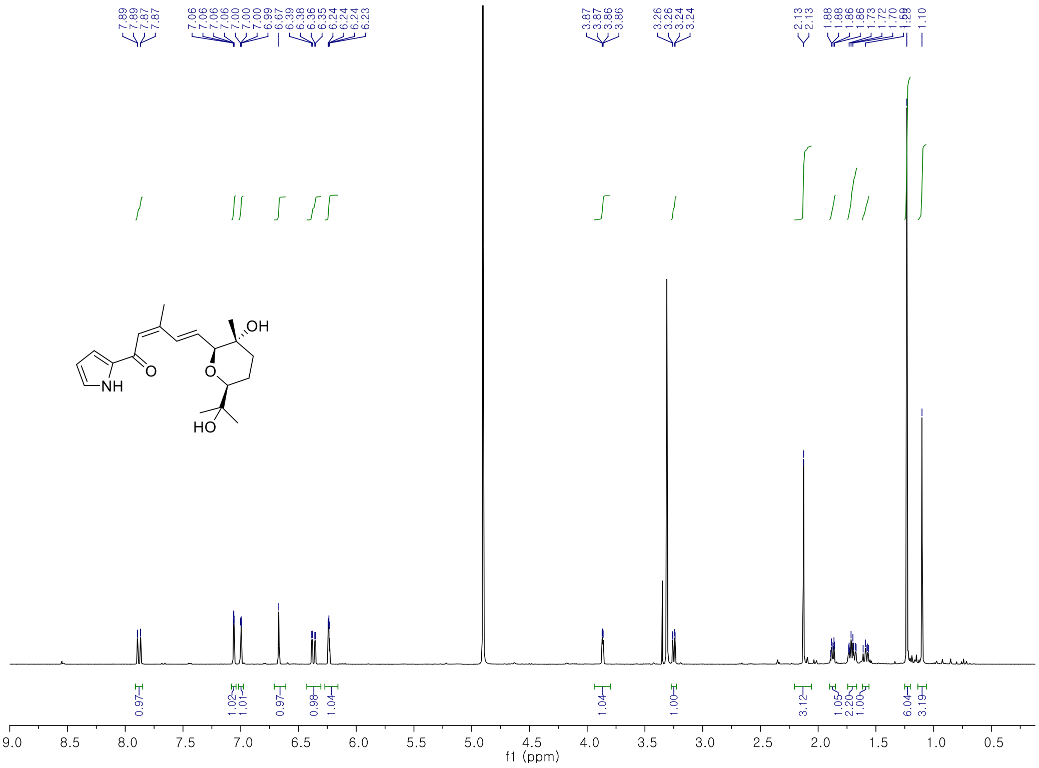

Figure S2. ^{13}C NMR spectrum (125 MHz) of glaciapyrrole D (**1**) in MeOH-d_4.

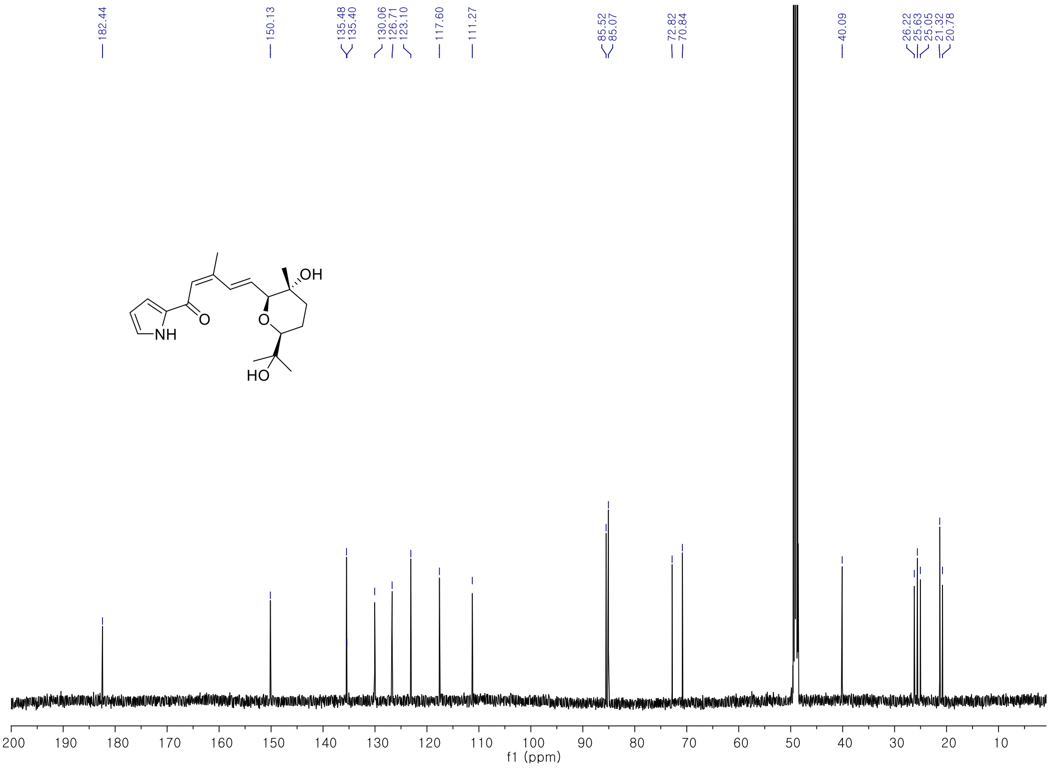

Figure S3. COSY spectrum (600 MHz) of glaciapyrrole D (**1**) in MeOH-d_4.

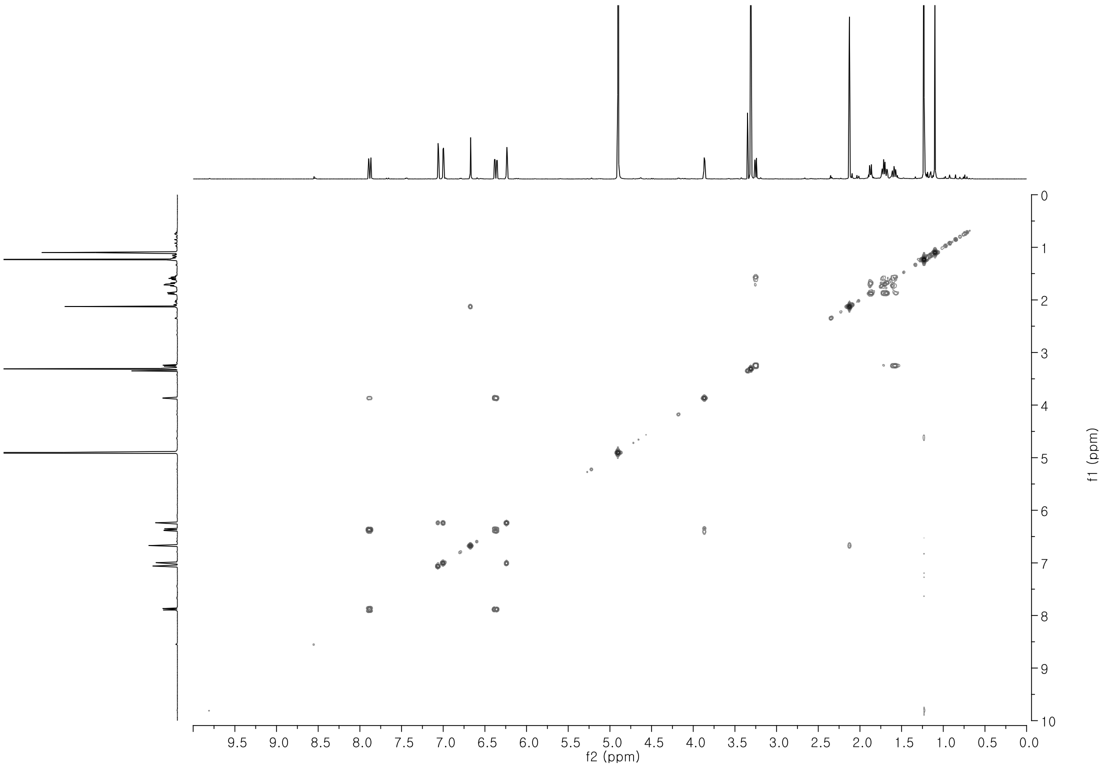

Figure S4. ROSEY spectrum (600 MHz) of glaciapyrrole D (**1**) in MeOH-d_4.

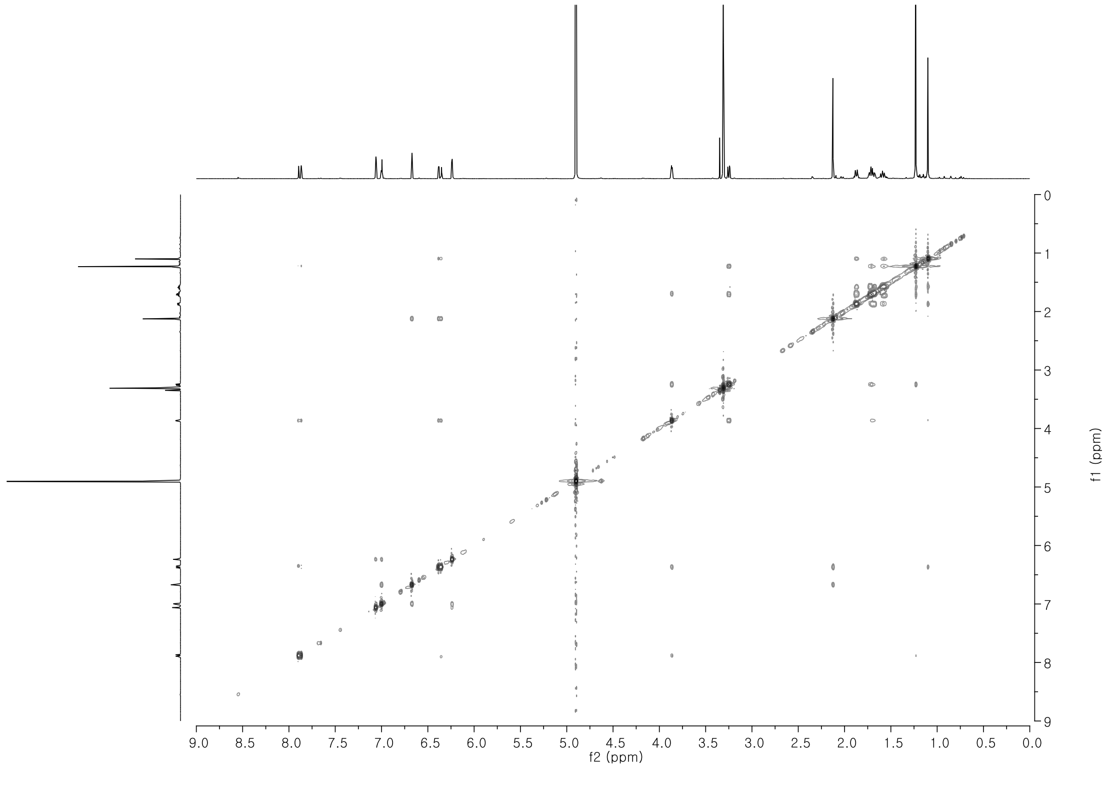

Figure S5. HSQC spectrum (600 MHz) of glaciapyrrole D (**1**) in MeOH-d_4.

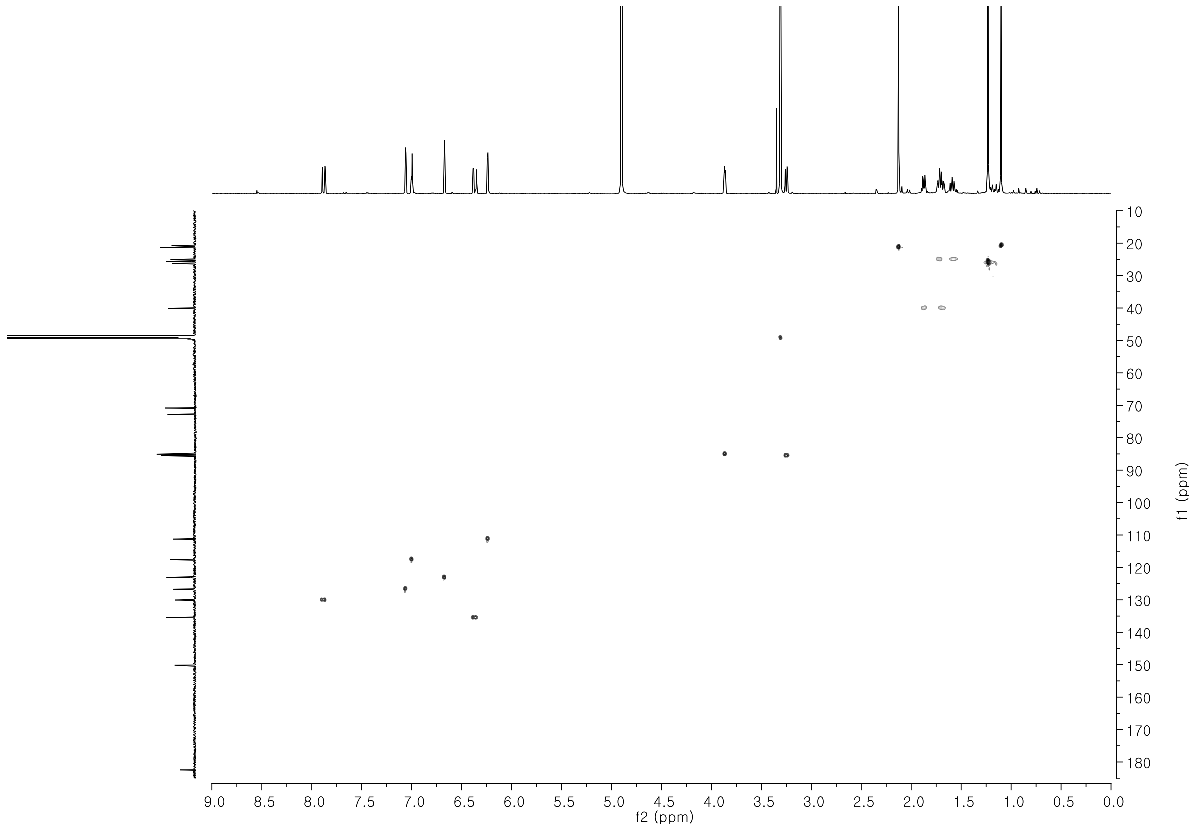

Figure S6. HMBC spectrum (600 MHz) of glaciapyrrole D (**1**) in MeOH-d_4.

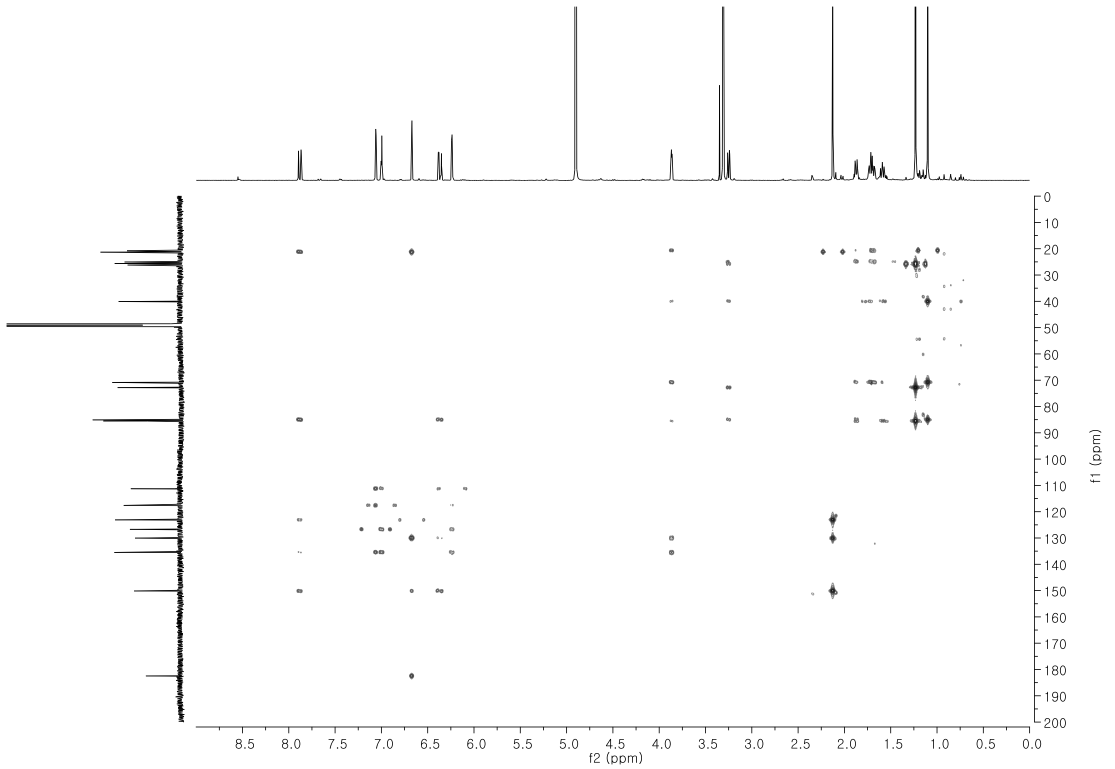

Figure S7. ^{1}H NMR spectrum (600 MHz) of glaciapyrrole E (**2**) in MeOH-d_4.

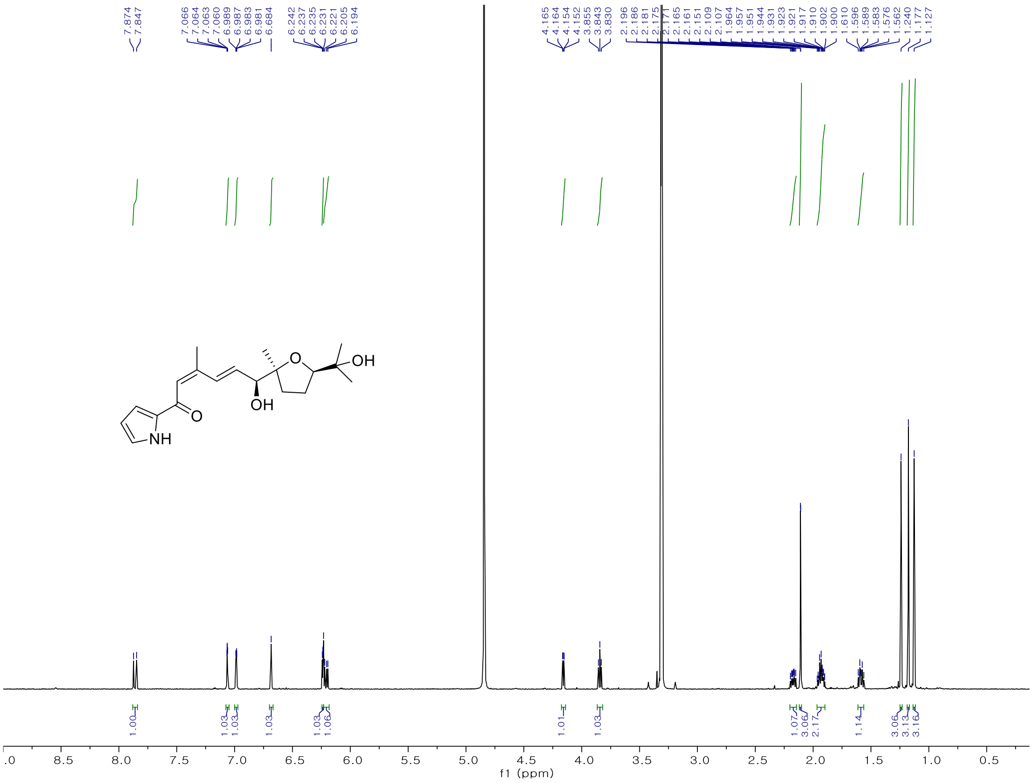

Figure S8. ^{13}C NMR spectrum (125 MHz) of glaciapyrrole E (**2**) in MeOH-d_4.

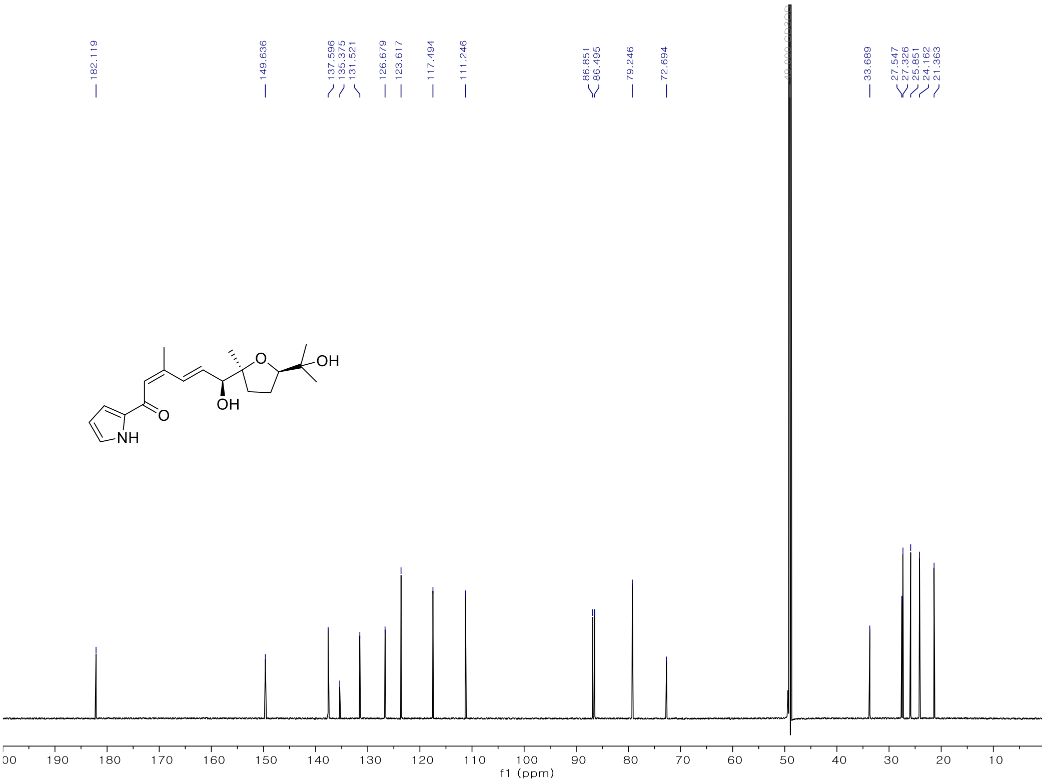

Figure S9. COSY spectrum (600 MHz) of glaciapyrrole E (**2**) in MeOH-d_4.

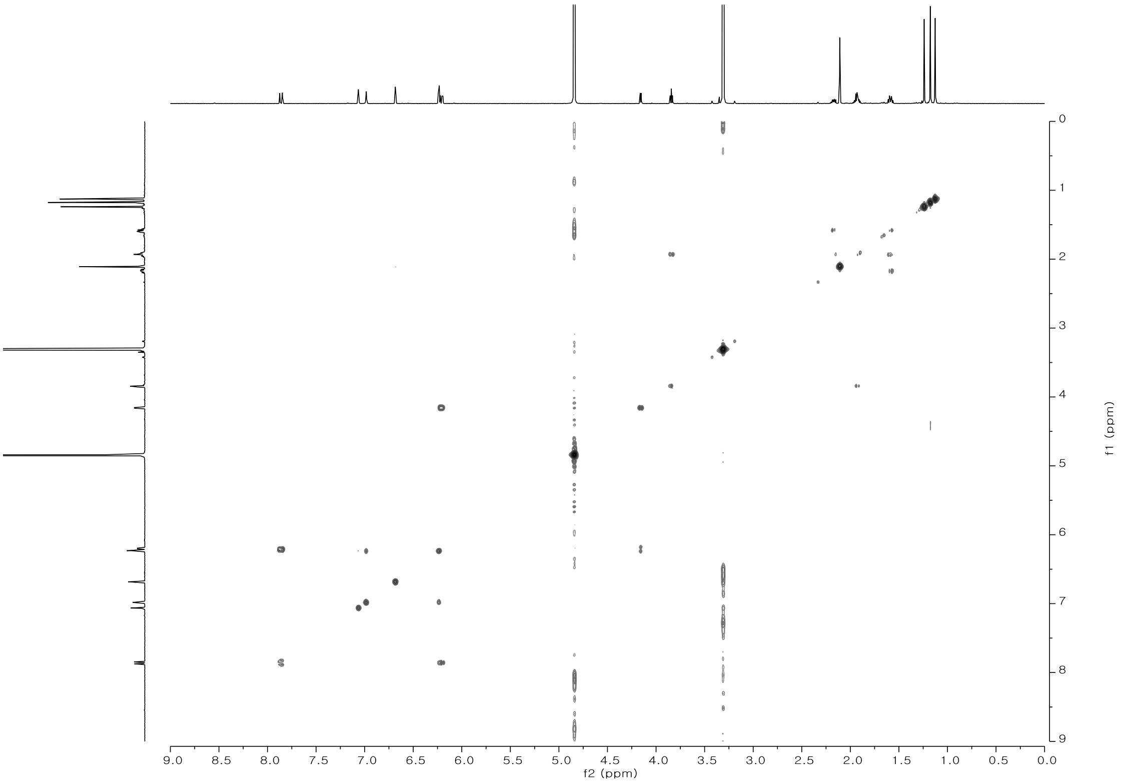

Figure S10. ROESY spectrum (600 MHz) of glaciapyrrole E (**2**) in MeOH-d_4.

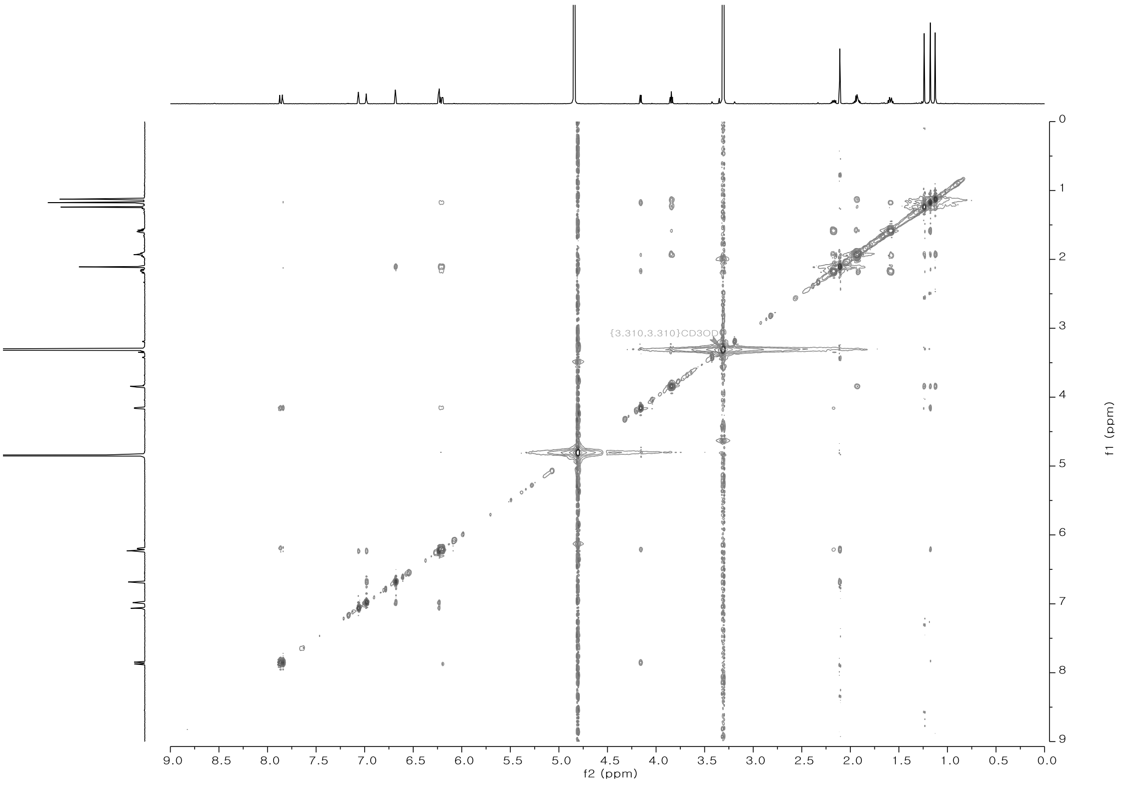

Figure S11. HSQC spectrum (600 MHz) of glaciapyrrole E (**2**) in MeOH-d_4.

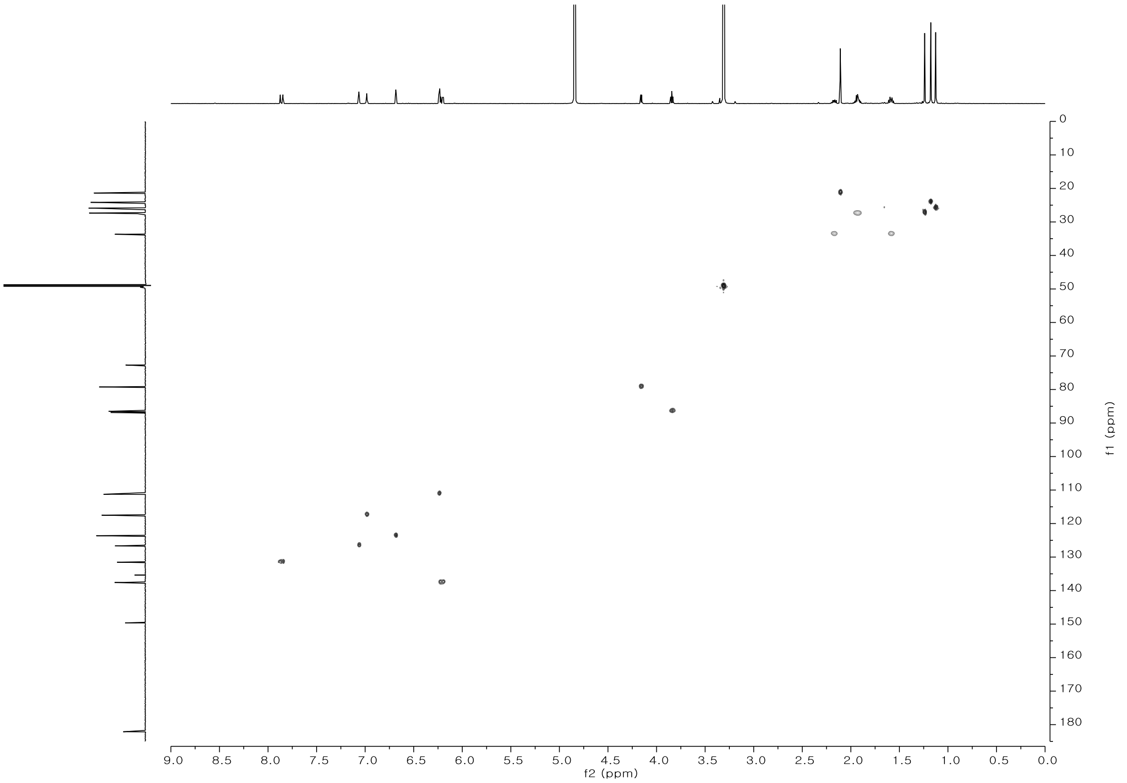

Figure S12. HMBC spectrum (600 MHz) of glaciapyrrole E (**2**) in MeOH-d_4.

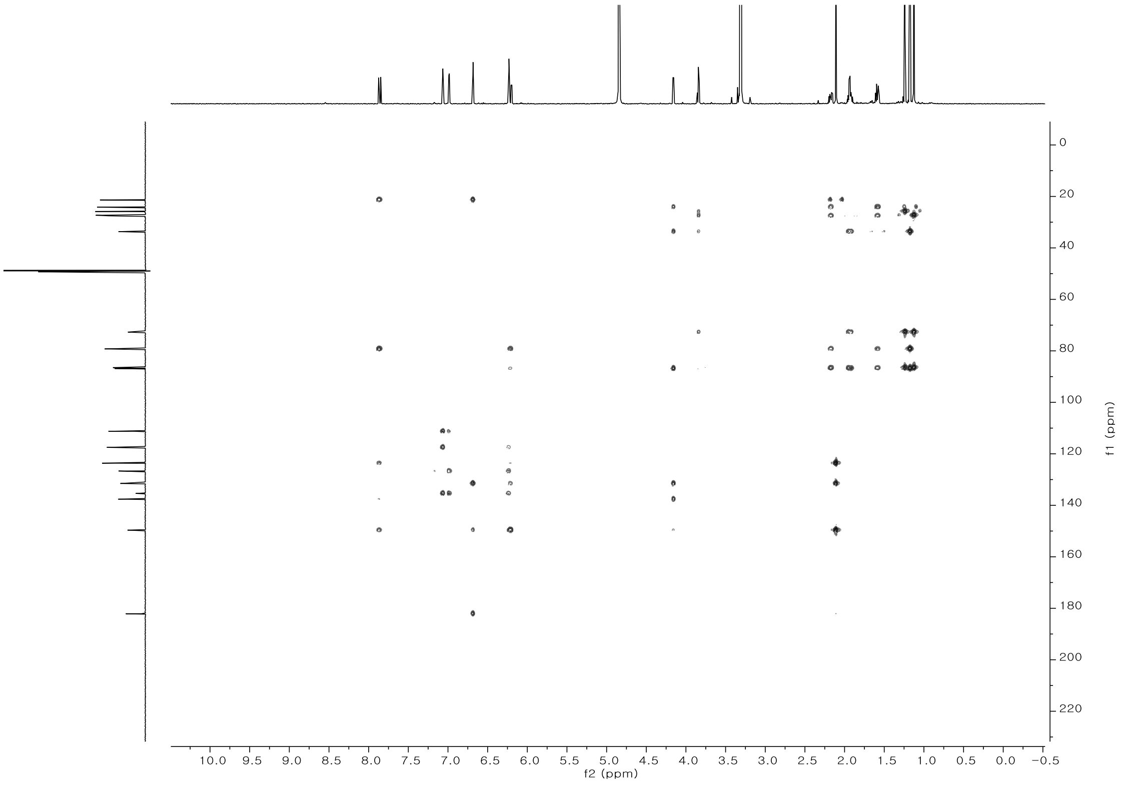

Figure S13. ^{1}H NMR spectrum (850 MHz) of photoglaciapyrrole D (**4**) in MeOH-d_4.

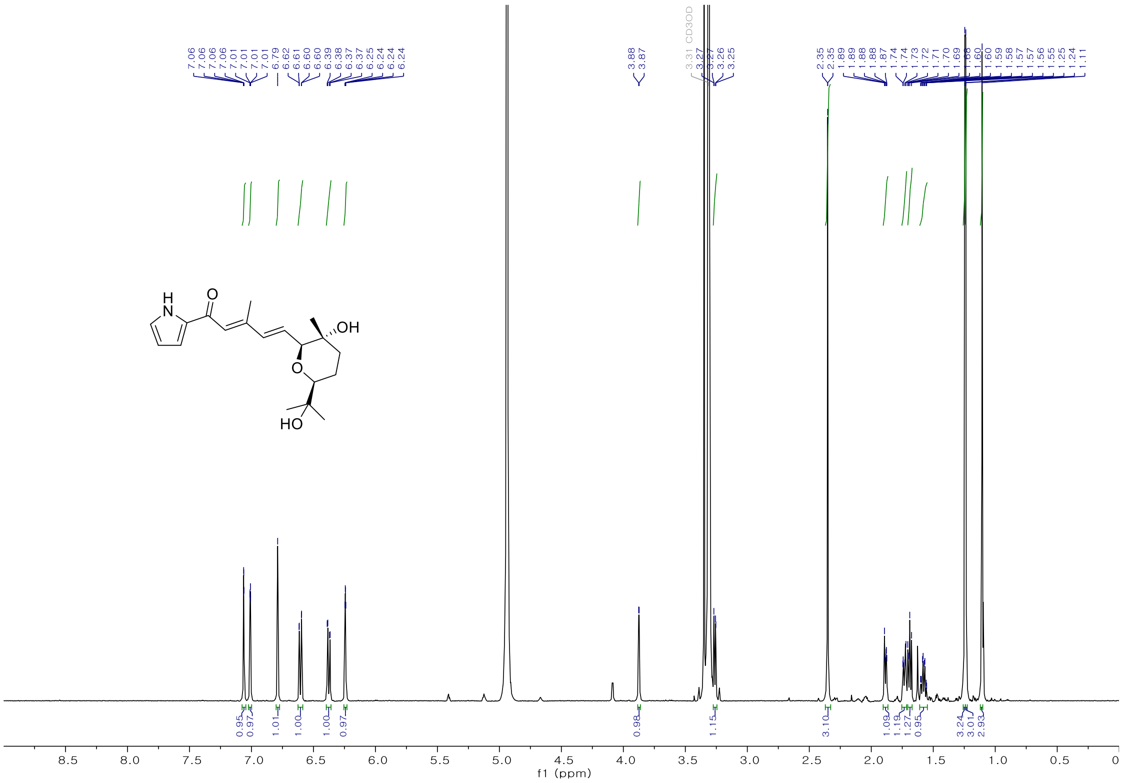

Figure S14. ^{13}C NMR spectrum (212.5 MHz) of photoglaciapyrrole D (**4**) in MeOH-d_4.

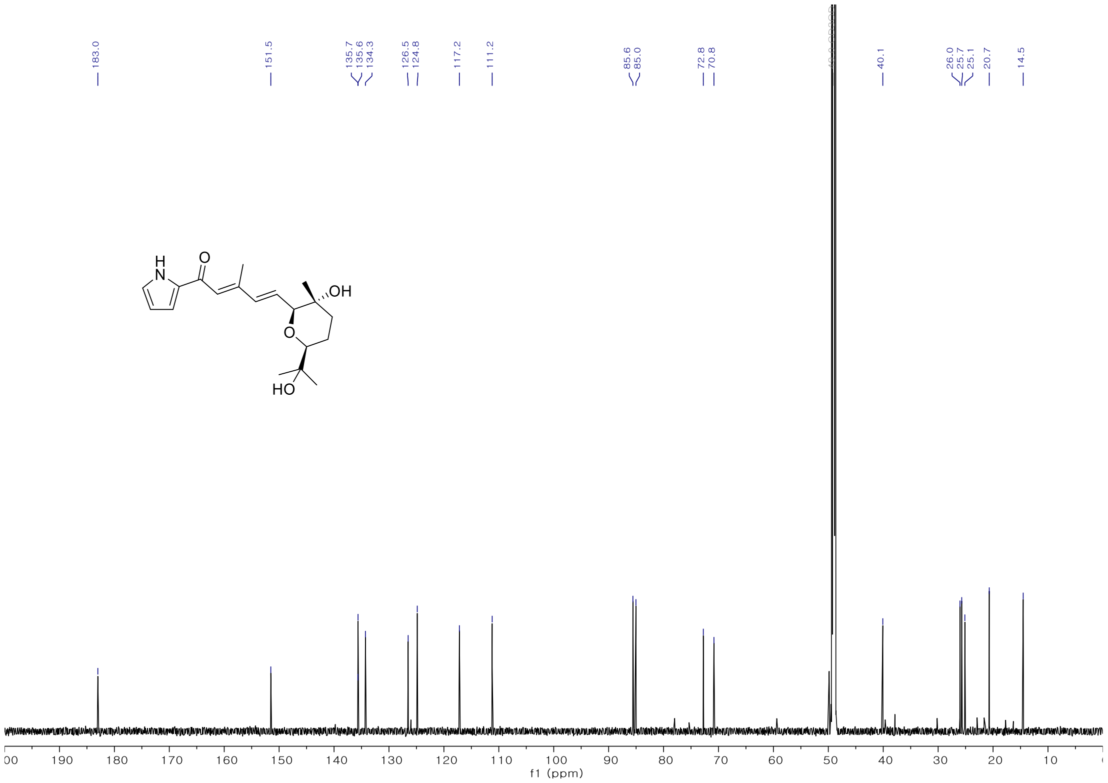

Figure S15. COSY spectrum (850 MHz) of photoglaciapyrrole D (**4**) in MeOH-d_4.

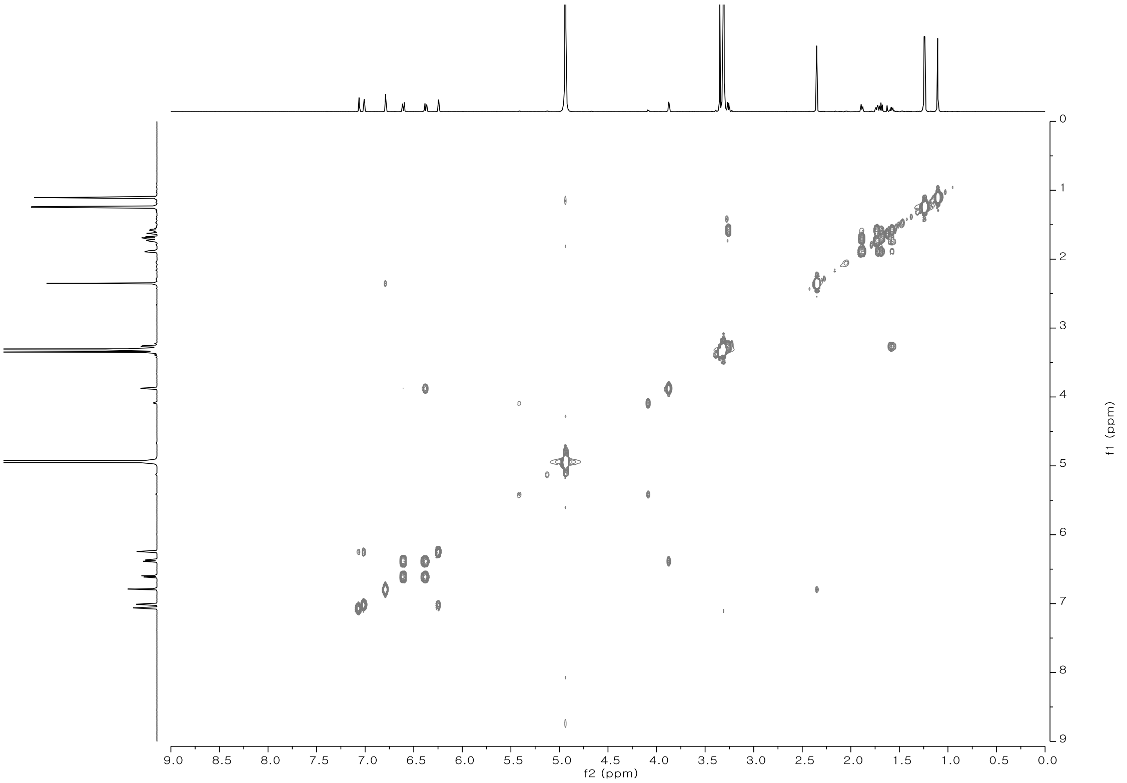

Figure S16. ROESY spectrum (850 MHz) of photoglaciapyrrole D (**4**) in MeOH-d_4.

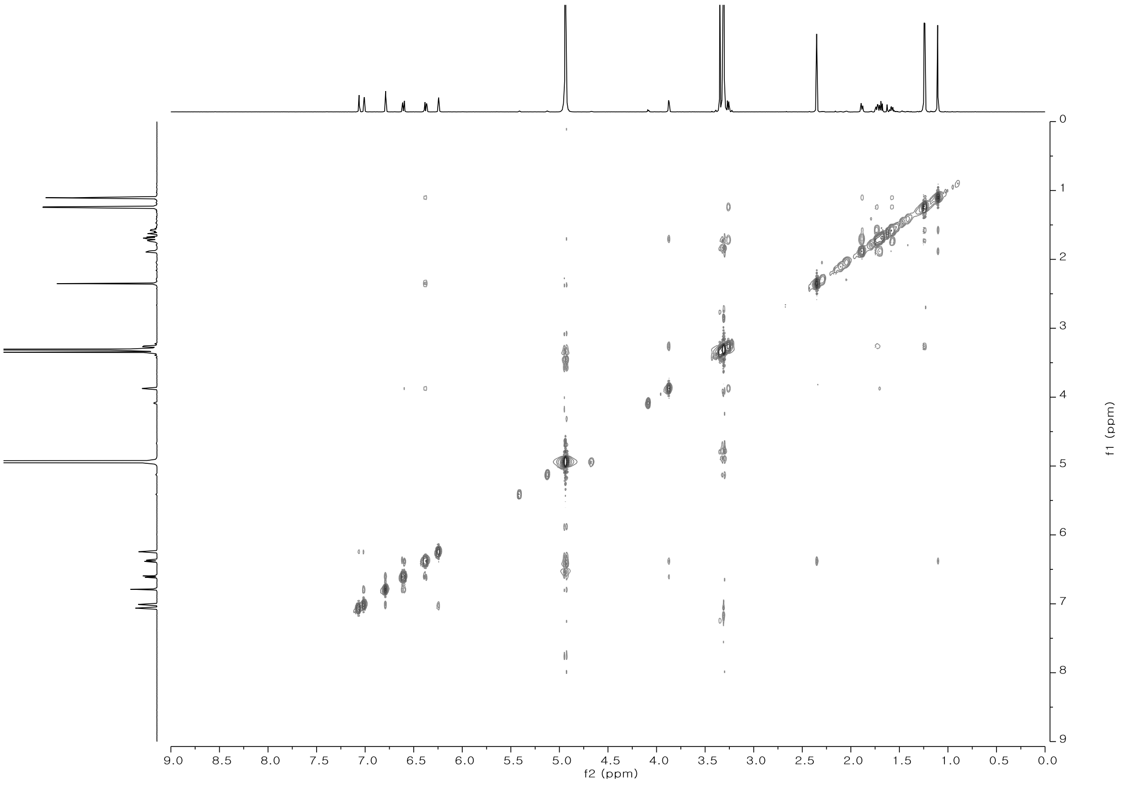

Figure S17. HSQC spectrum (850 MHz) of photoglaciapyrrole D (**4**) in MeOH-d_4.

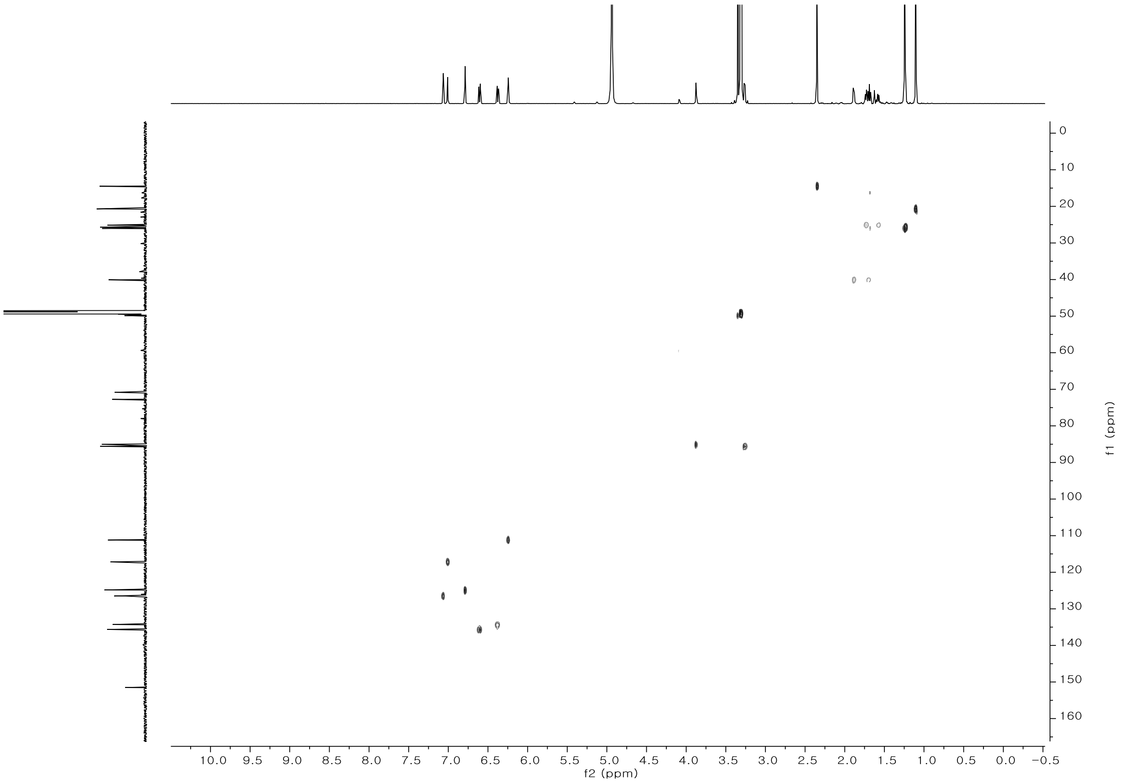

Figure S18. HMBC spectrum (850 MHz) of photoglaciapyrrole D (**4**) in MeOH-d_4.

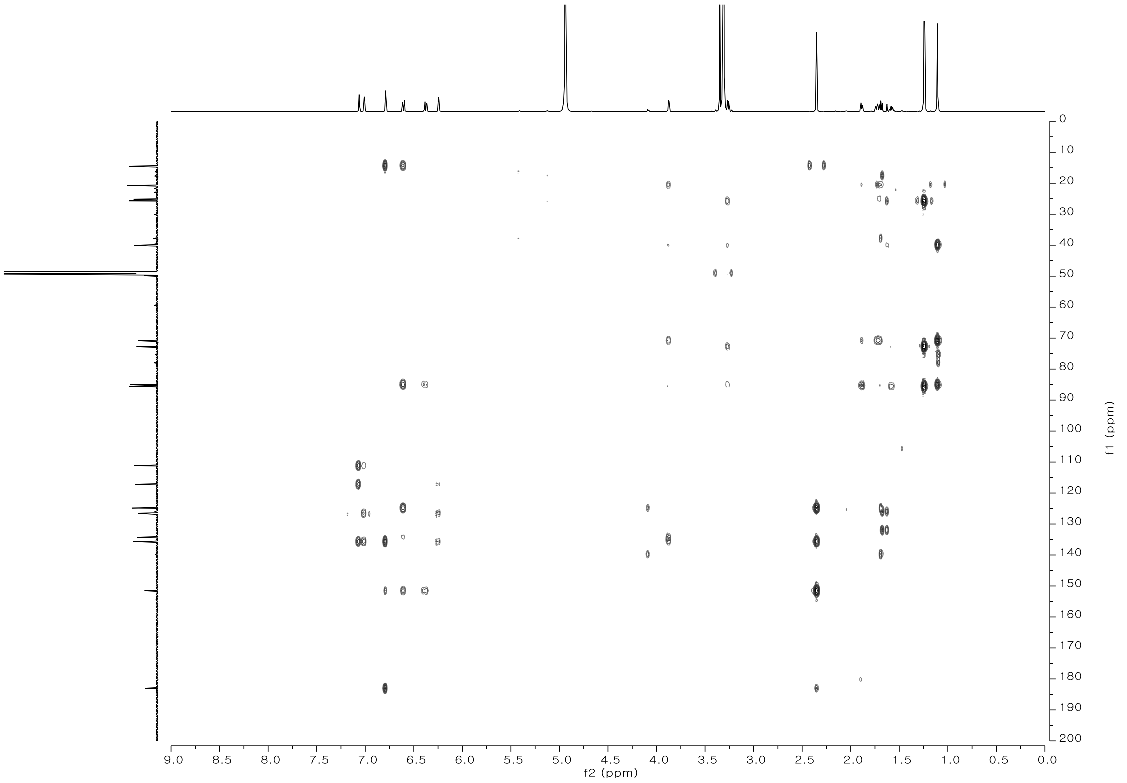

Figure S19. ^{1}H NMR spectrum (850 MHz) of photoglaciapyrrole E (**5**) in MeOH-d_4.

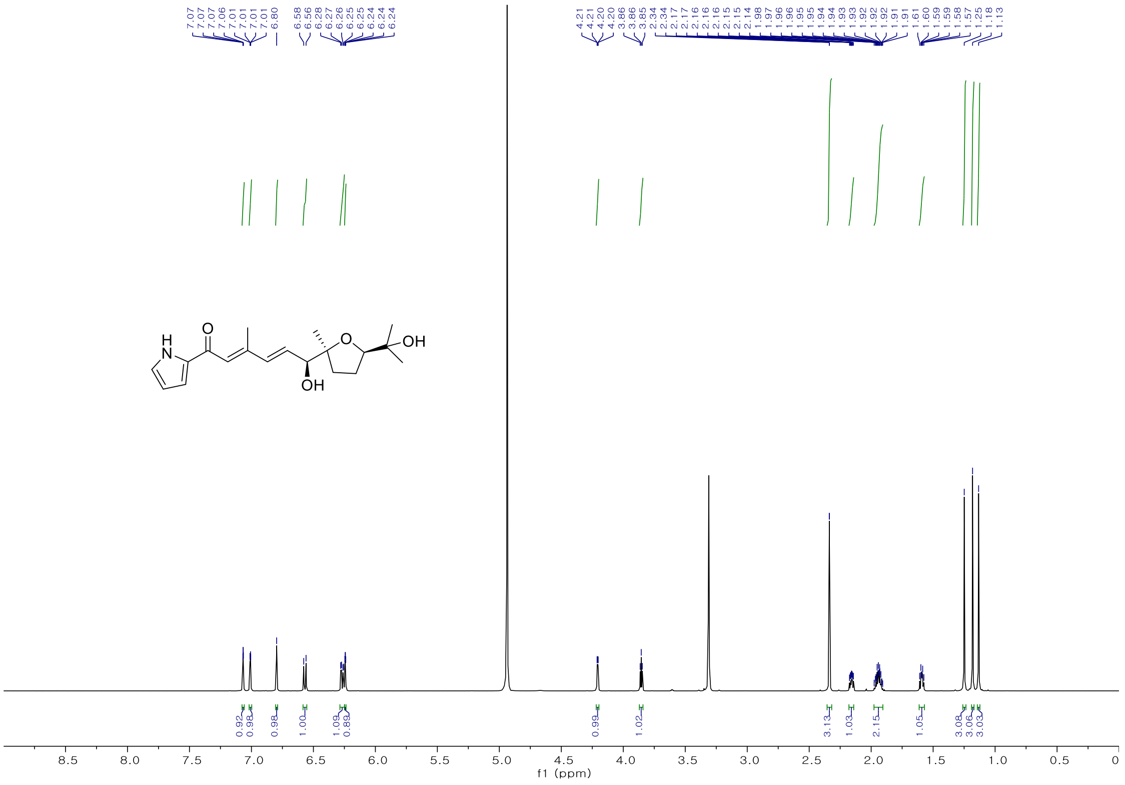

Figure S20. ^{13}C NMR spectrum (212.5 MHz) of photoglaciapyrrole E (**5**) in MeOH-d_4.

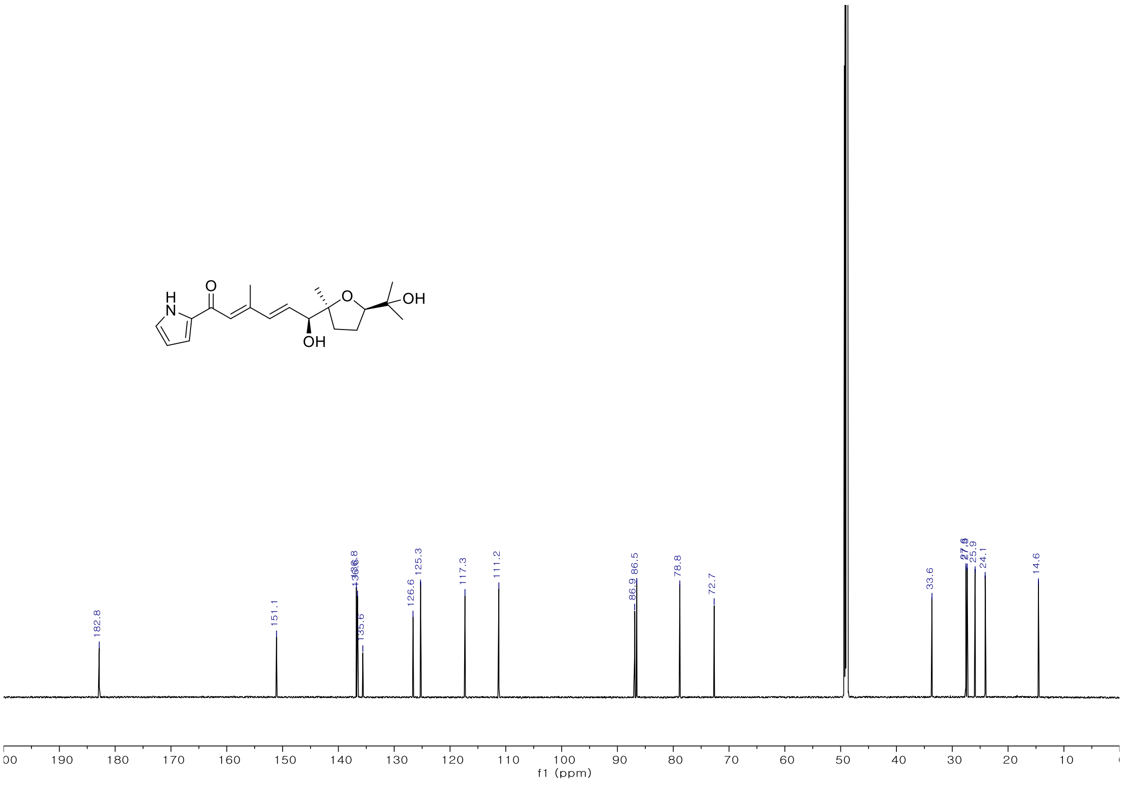

Figure S21. COSY spectrum (850 MHz) of photoglaciapyrrole E (**5**) in MeOH-d_4.

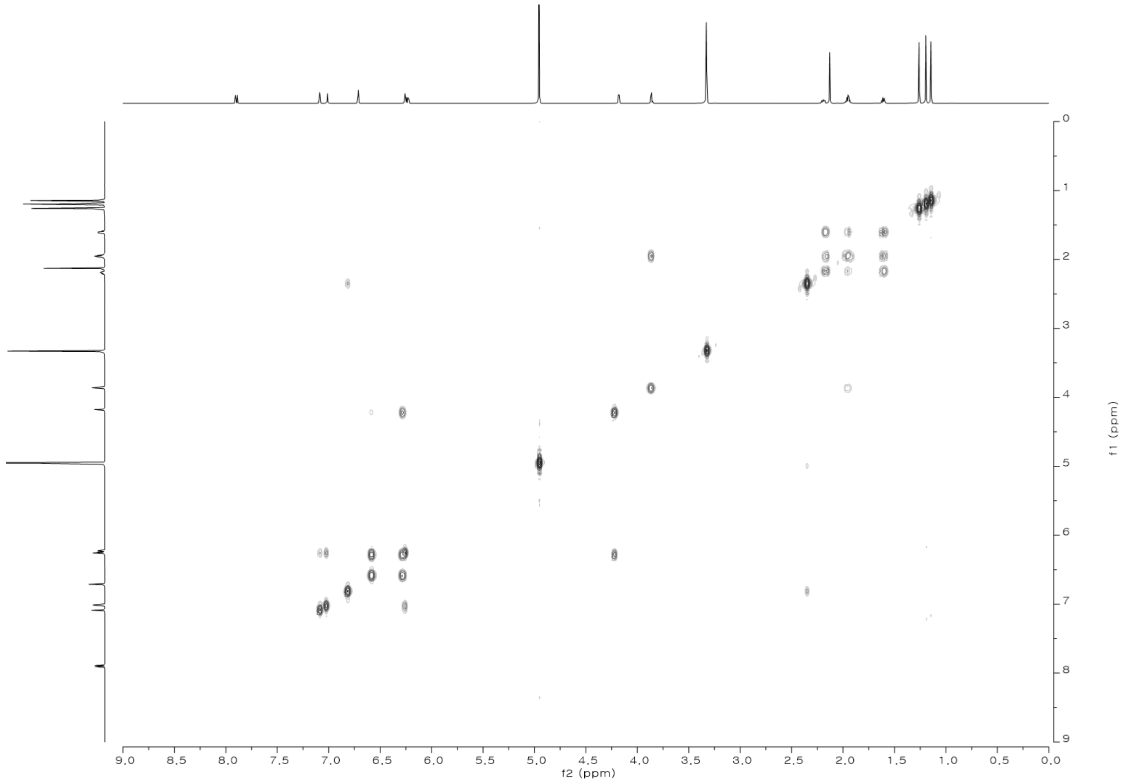

Figure S22. ROESY spectrum (850 MHz) of photoglaciapyrrole E (**5**) in MeOH-d_4.

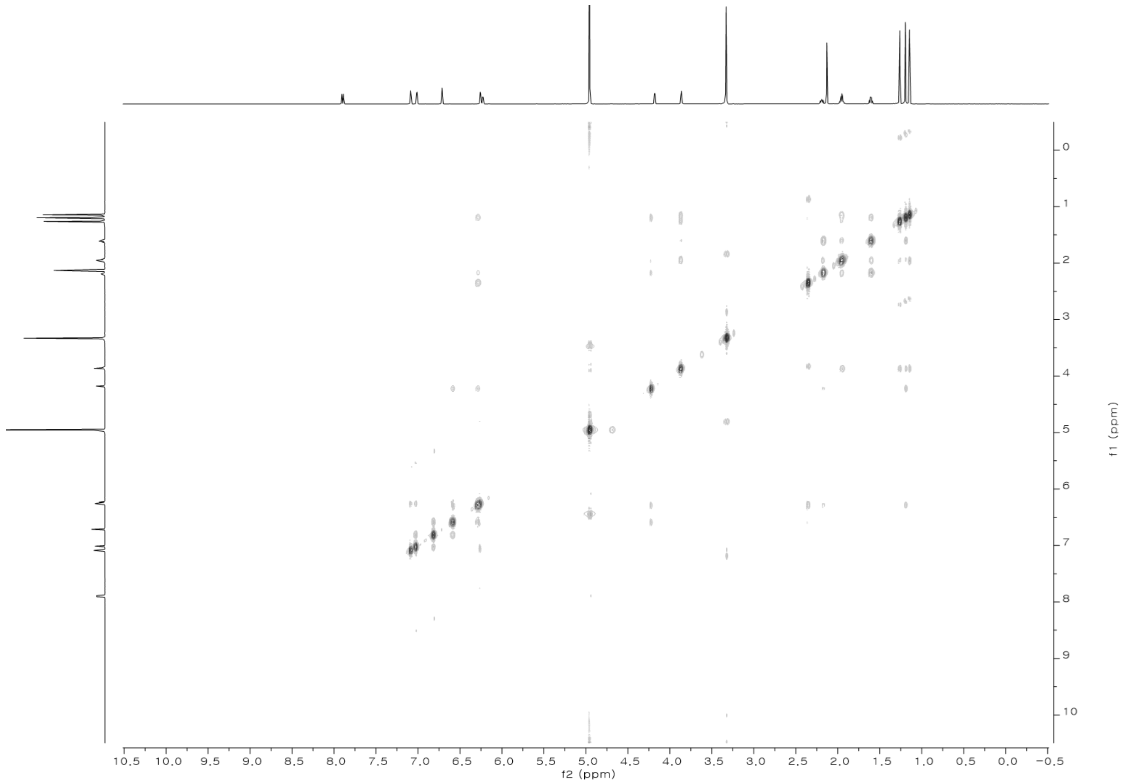

Figure S23. HSQC spectrum (850 MHz) of photoglaciapyrrole E (**5**) in MeOH-d_4.

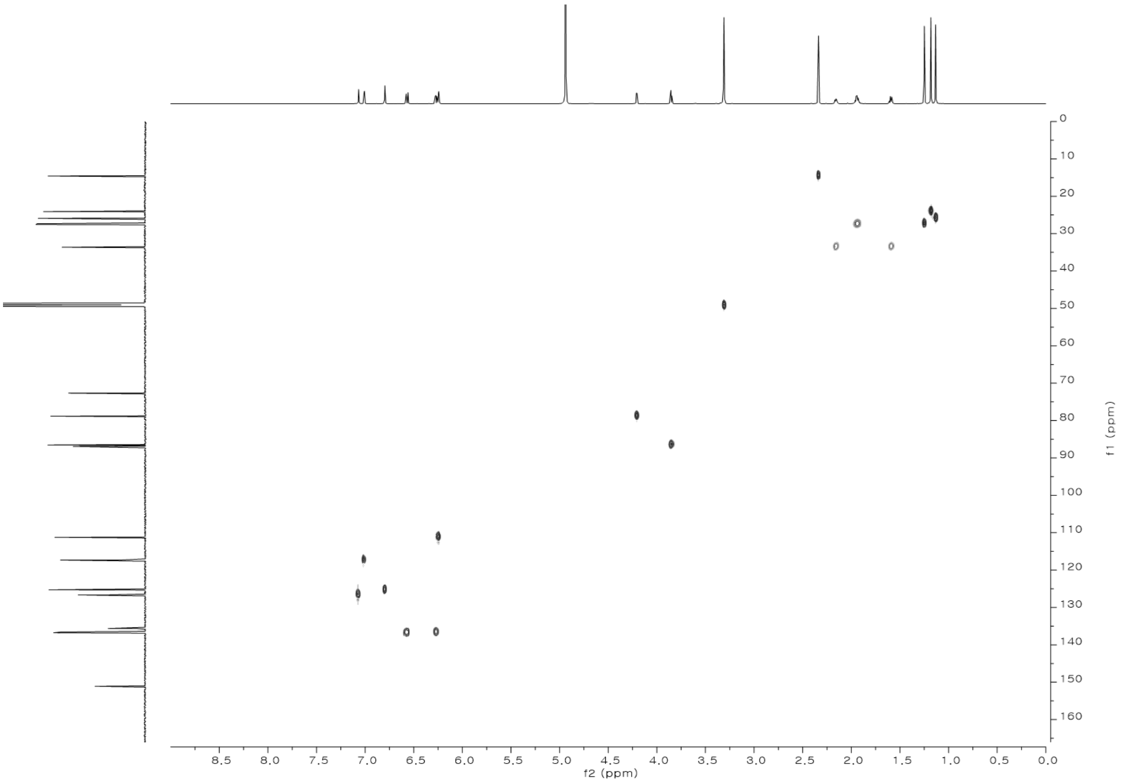

Figure S24. HMBC spectrum (850 MHz) of photoglaciapyrrole E (**5**) in MeOH-d_4.

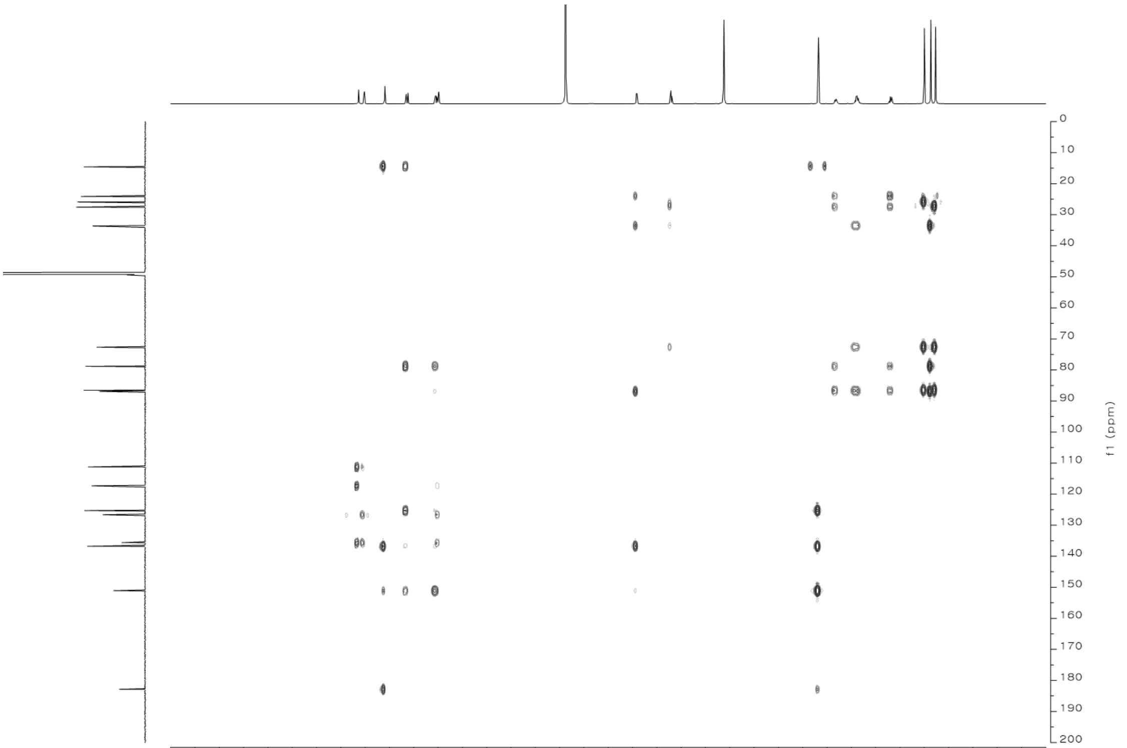

Figure S25. ^{1}H NMR spectrum (850 MHz) of photoglaciapyrrole A (**6**) in MeOH-d_4.

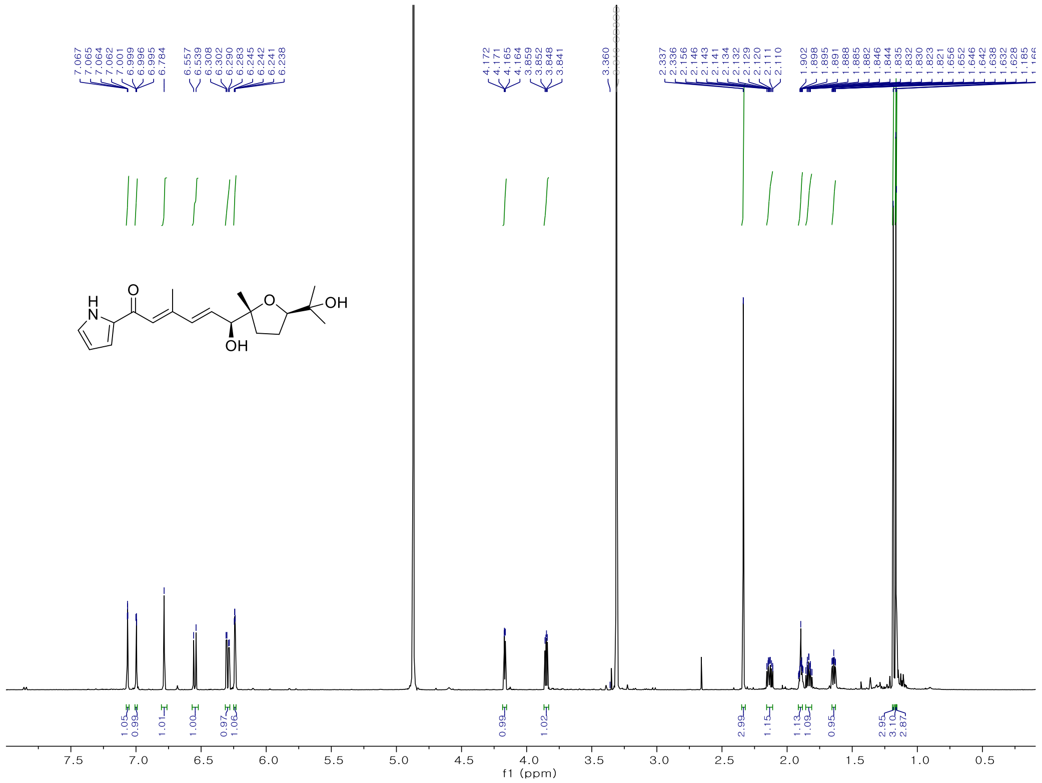

Figure S26. ^{13}C NMR spectrum (212.5 MHz) of photoglaciapyrrole A (**6**) in MeOH-d_4.

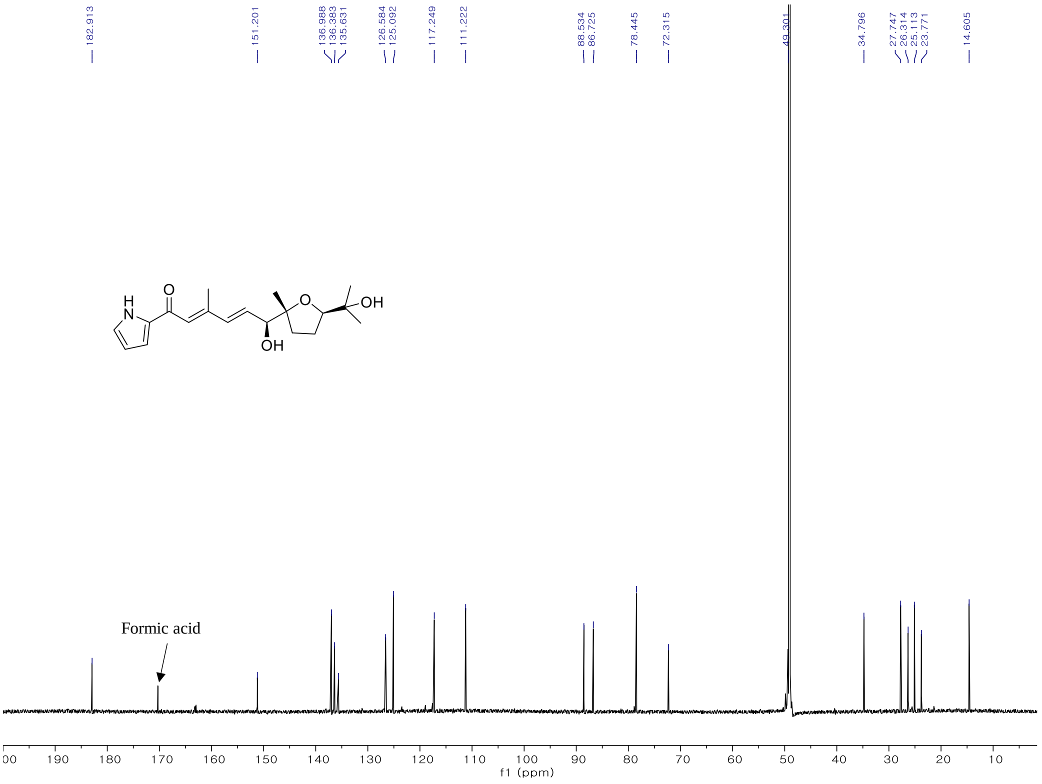

Figure S27. COSY spectrum (850 MHz) of photoglaciapyrrole A (**6**) in MeOH-d_4.

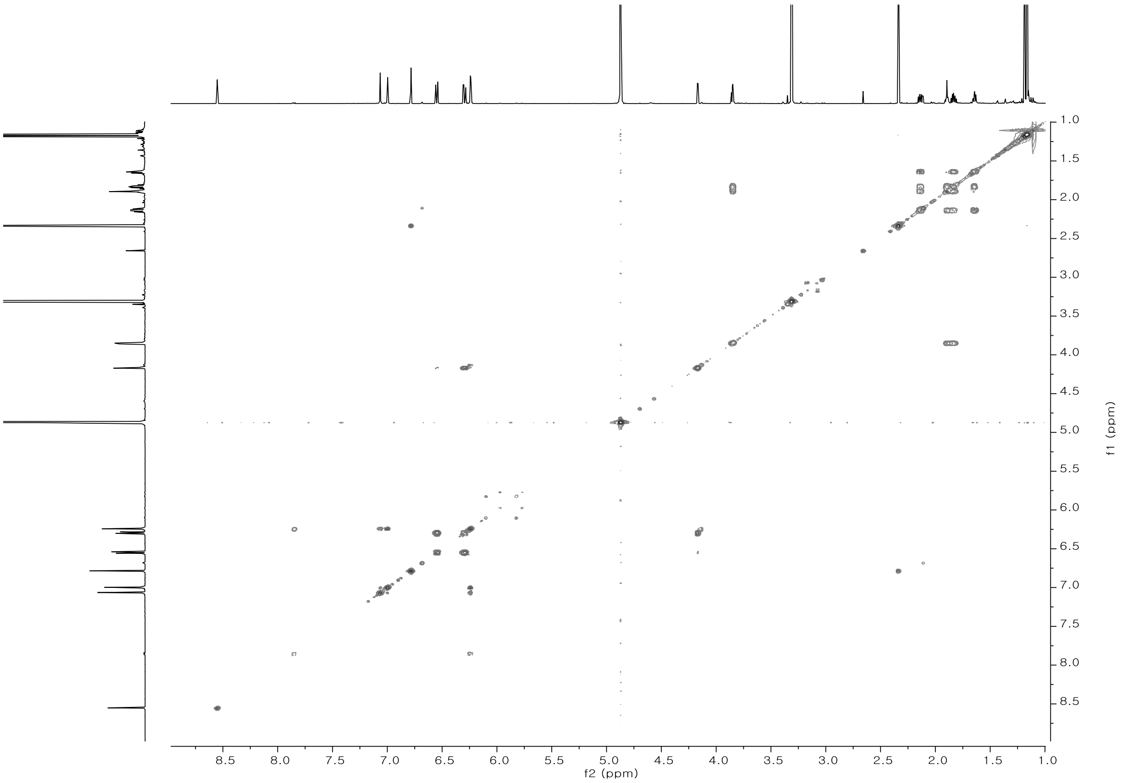

Figure S28. ROESY spectrum (850 MHz) of photoglaciapyrrole A (**6**) in MeOH-d_4.

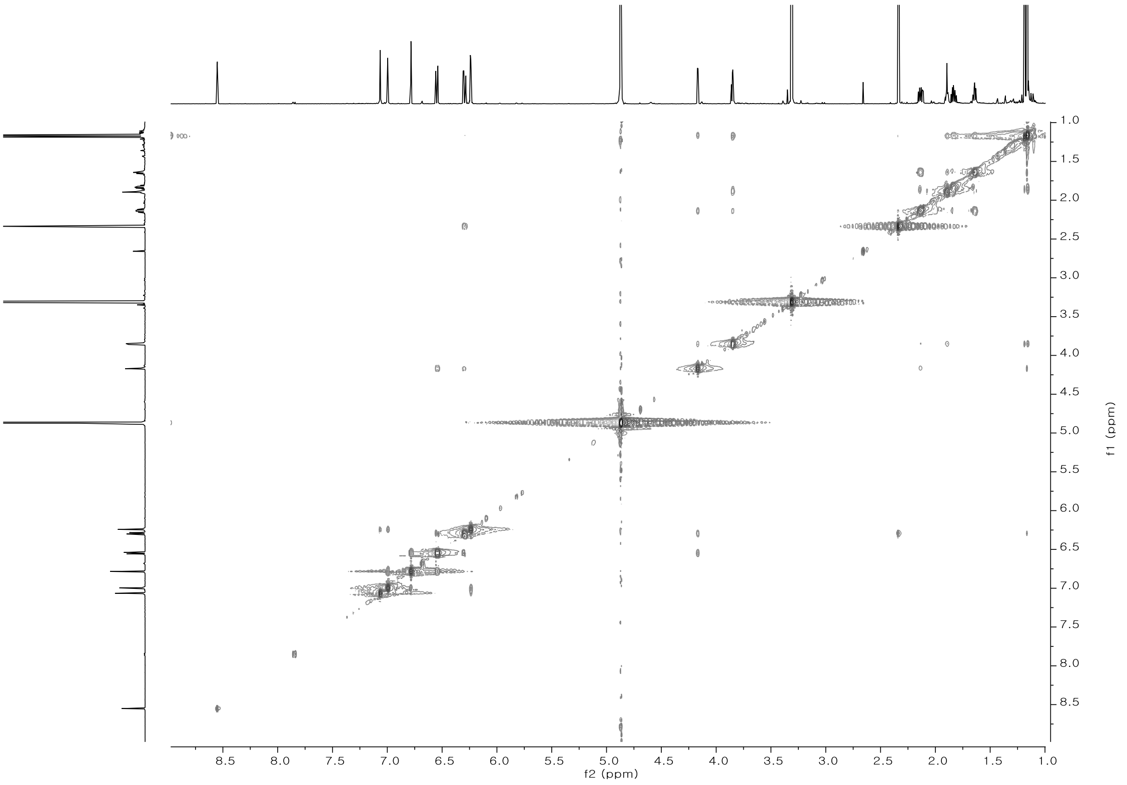

Figure S29. HSQC spectrum (850 MHz) of photoglaciapyrrole A (**6**) in MeOH-d_4.

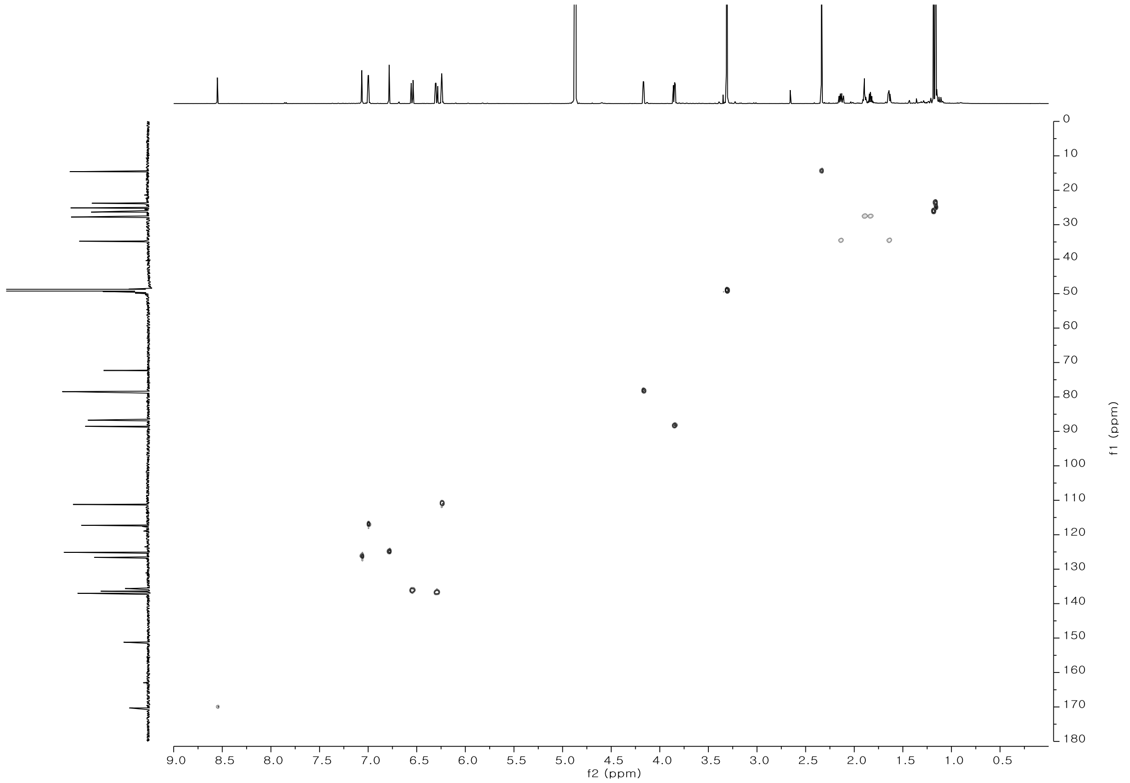

Figure S30. HMBC spectrum (850 MHz) of photoglaciapyrrole A (**6**) in MeOH-d_4.

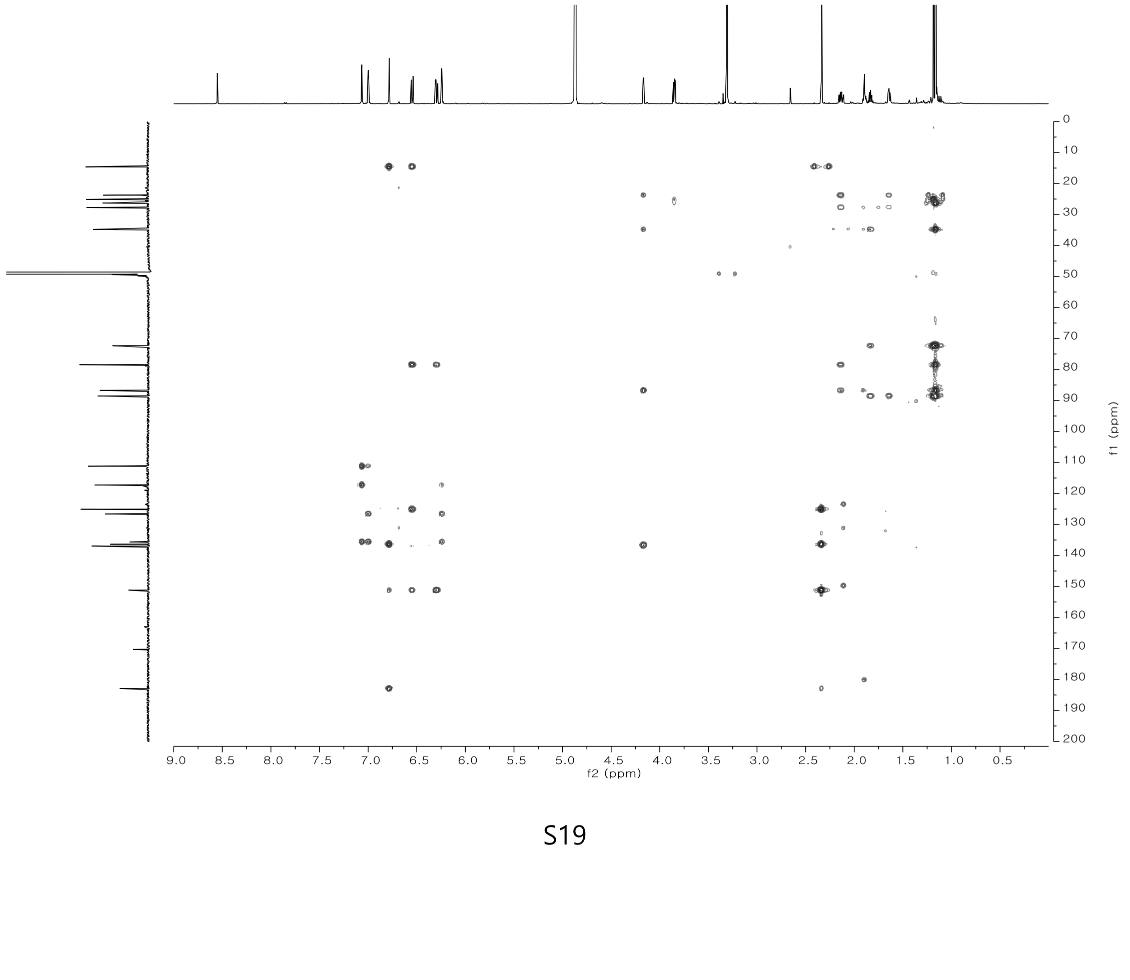

Figure S31. ^{1}H NMR spectrum (600 MHz) of glaciapyrrole A (**3**) in MeOH-d_4.

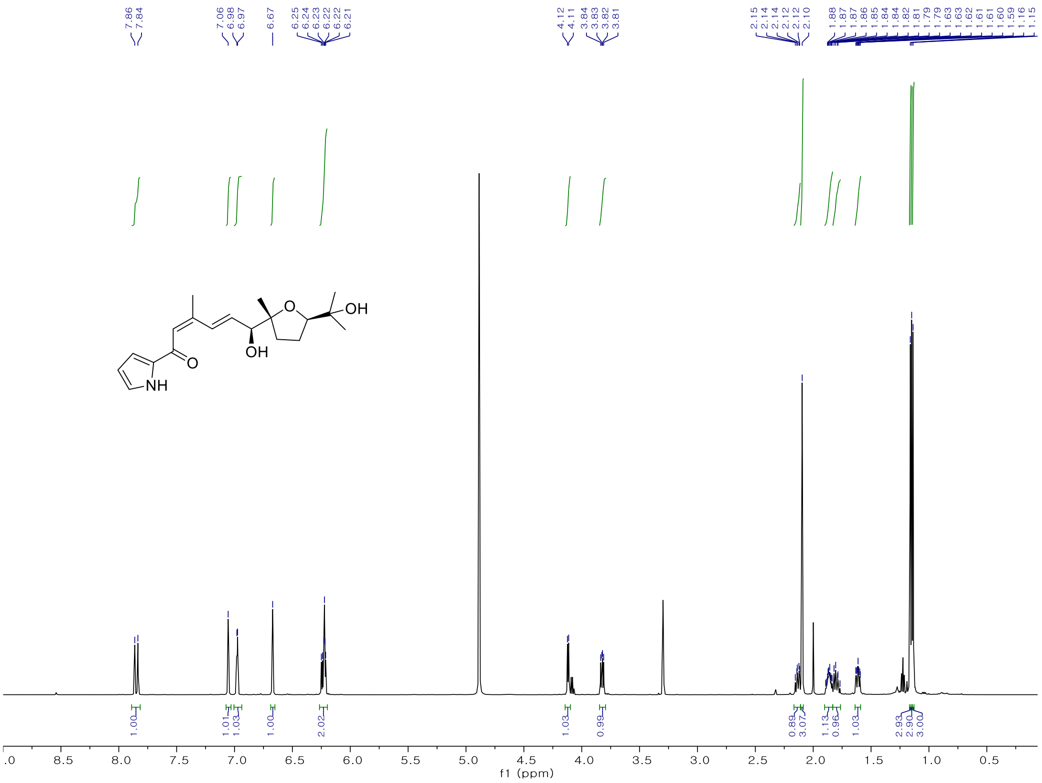

Figure S32. ^{1}H NMR spectrum (500 MHz) of *S*-MTPA ester (**7**) for glaciapyrrole E (**2**) in MeOH-d_4.

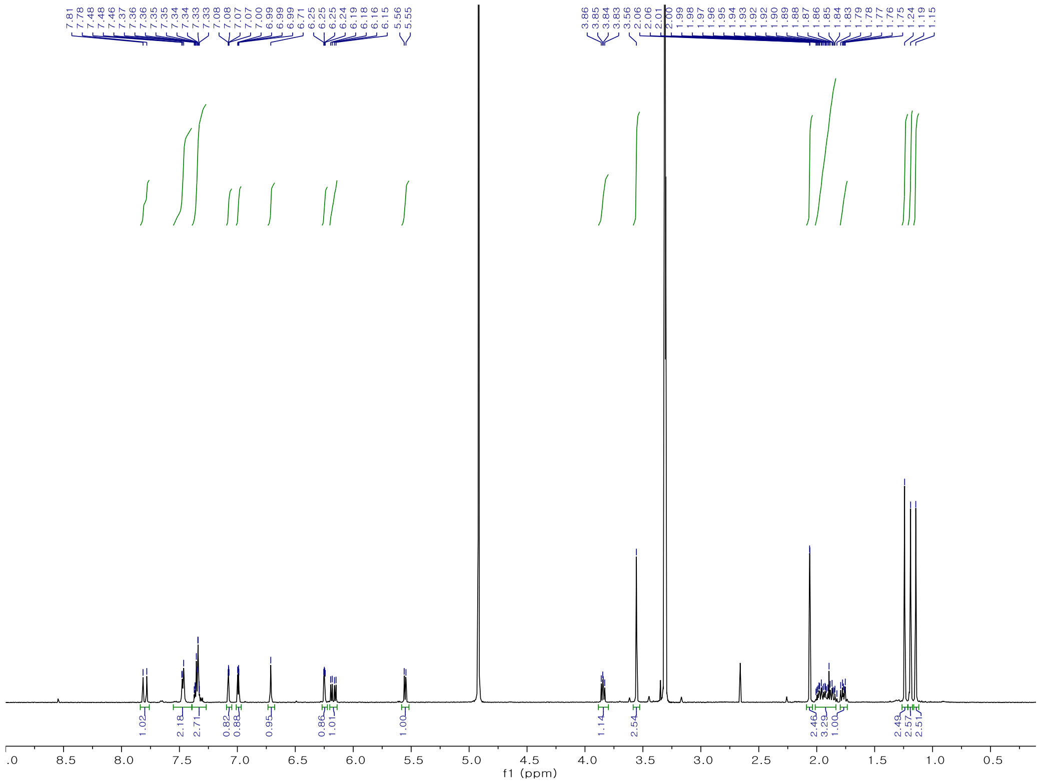

Figure S33. COSY NMR spectrum (500 MHz) of *S*-MTPA ester (**7**) for glaciapyrrole E (**2**) in MeOH-d_4.

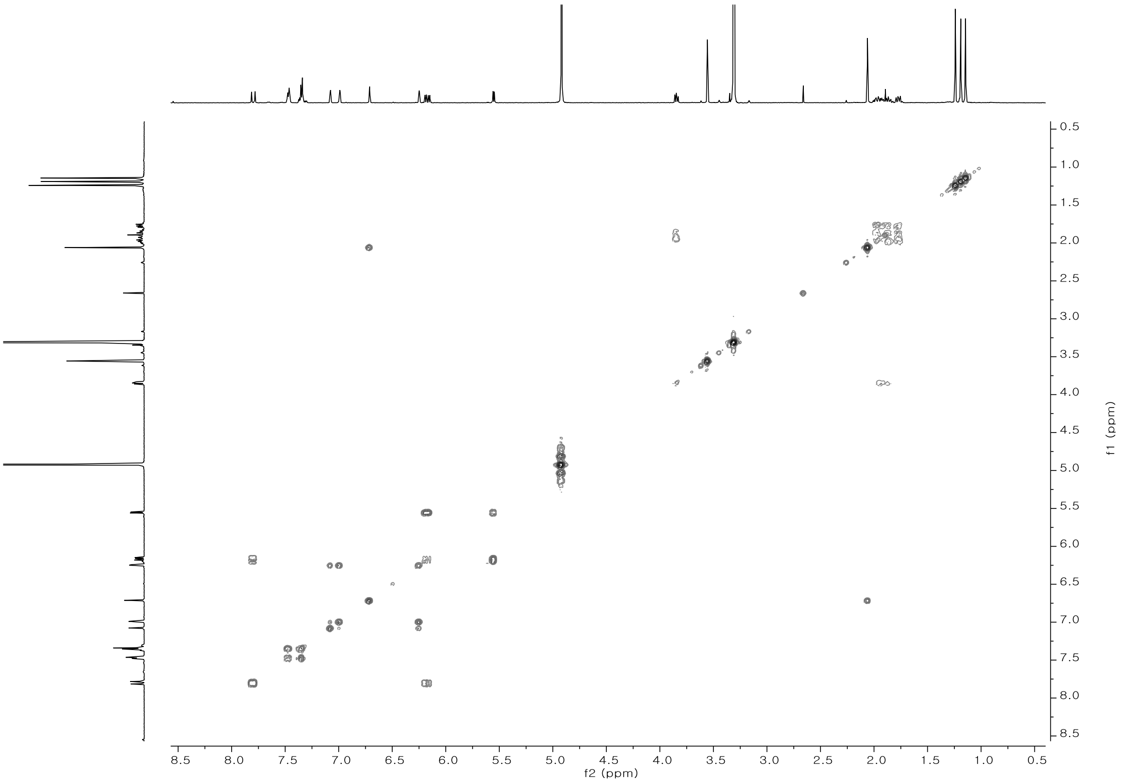

Figure S34. ^{1}H NMR spectrum (500 MHz) of *R*-MTPA ester (**8**) for glaciapyrrole E (**2**) in MeOH-d_4.

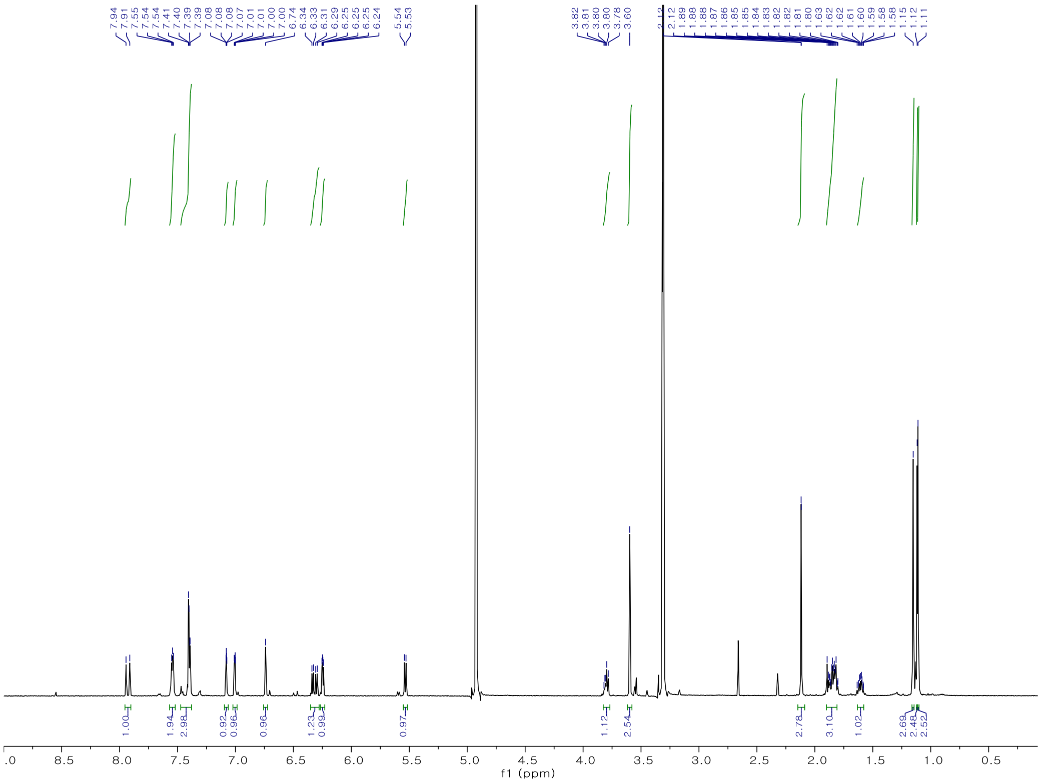

Figure S35. COSY NMR spectrum (500 MHz) of *R*-MTPA ester (**8**) for glaciapyrrole E (**2**) in MeOH-d_4.

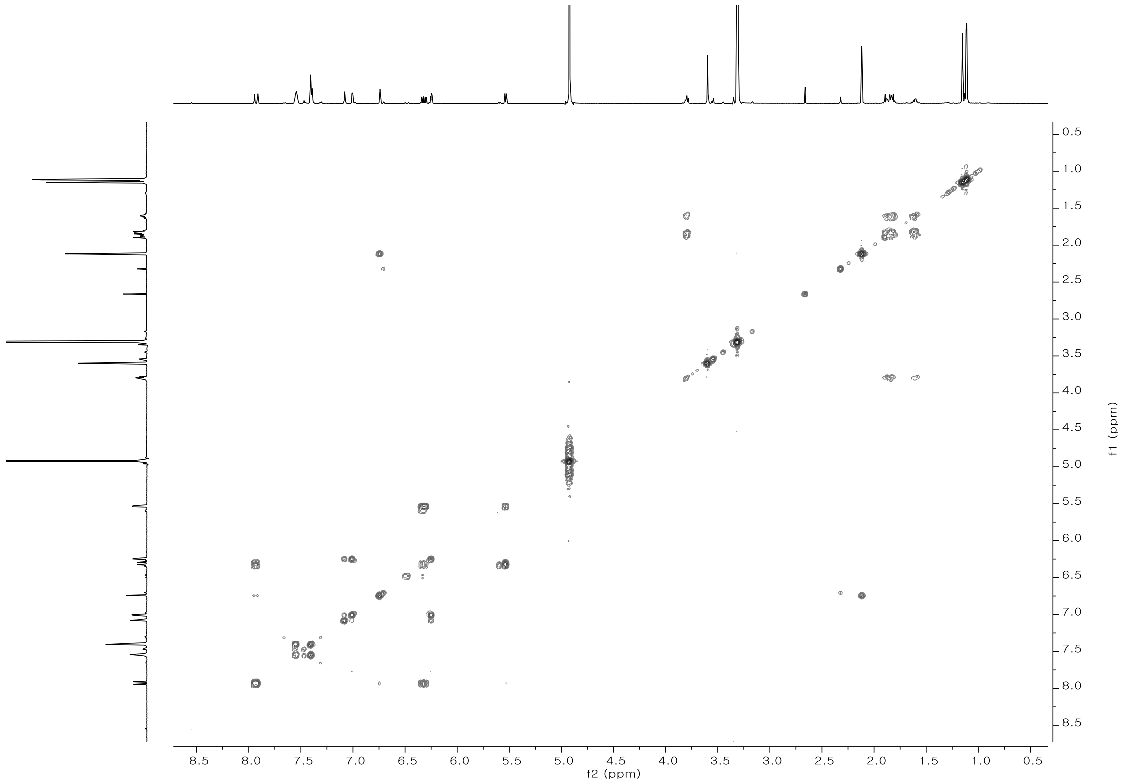

Figure S36. HR-FAB-MS data of glaciapyrrole D (**1**)

[Mass Spectrum]
Data : FAB-B048 Date : 09-Sep-2020 15:26
RT : 0.99 min Scan# : (36,57)
Elements : C 100/0, H 100/0, N 10/0, O 10/0
Mass Tolerance : 1000ppm, 5mmu if m/z < 5, 20mmu if m/z > 20
Unsaturation (U.S.) : 0.0 – 20.0

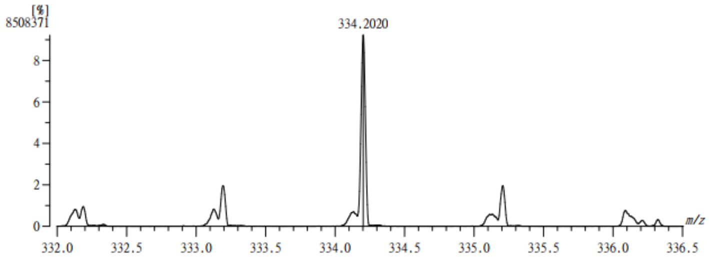

	Observed m/z	Int%	Err[ppm / mmu]	U.S.	Composition
1	334.2020	9.22	-41.1 / -13.7	11.0	C21 H26 N4
2			-3.5 / -1.2	11.5	C20 H24 N5
3			+34.1 / +11.4	12.0	C19 H22 N6
4			-45.2 / -15.1	10.5	C23 H28 N O
5			-7.5 / -2.5	11.0	C22 H26 N2 O
6			+30.1 / +10.1	11.5	C21 H24 N3 O
7			-25.1 / -8.4	7.5	C14 H24 N9 O
8			+12.6 / +4.2	8.0	C13 H22 N10 O
9			+26.1 / +8.7	11.0	C23 H26 O2
10			-29.1 / -9.7	7.0	C16 H26 N6 O2
11			+8.5 / +2.9	7.5	C15 H24 N7 O2
12			+46.2 / +15.4	8.0	C14 H22 N8 O2
13			-33.1 / -11.1	6.5	C18 H28 N3 O3
14			+4.5 / +1.5	7.0	C17 H26 N4 O3
15			+42.1 / +14.1	7.5	C16 H24 N5 O3
16			-50.7 / -16.9	3.0	C10 H26 N10 O3
17			-37.1 / -12.4	6.0	C20 H30 O4
18			+0.5 / +0.2	6.5	C19 H28 N O4
19			+38.1 / +12.7	7.0	C18 H26 N2 O4
20			-54.7 / -18.3	2.5	C12 H28 N7 O4
21			-17.1 / -5.7	3.0	C11 H26 N8 O4
22			+20.6 / +6.9	3.5	C10 H24 N9 O4
23			+58.2 / +19.5	4.0	C9 H22 N10 O4
24			-58.7 / -19.6	2.0	C14 H30 N4 O5
25			-21.1 / -7.0	2.5	C13 H28 N5 O5
26			+16.6 / +5.5	3.0	C12 H26 N6 O5
27			+54.2 / +18.1	3.5	C11 H24 N7 O5
28			-25.1 / -8.4	2.0	C15 H30 N2 O6
29			+12.5 / +4.2	2.5	C14 H28 N3 O6
30			+50.2 / +16.8	3.0	C13 H26 N4 O6
31			+8.5 / +2.8	2.0	C16 H30 O7
32			+46.1 / +15.4	2.5	C15 H28 N O7

Figure S37. HR-FAB-MS data of glaciapyrrole E (**2**)

[Mass Spectrum]
Data : FAB-B049 Date : 09-Sep-2020 15:37
RT : 1.10 min Scan# : (40,44)
Elements : C 100/0, H 100/0, N 10/0, O 10/0
Mass Tolerance : 1000ppm, 5mmu if m/z < 5, 20mmu if m/z > 20
Unsaturation (U.S.) : 0.0 - 20.0

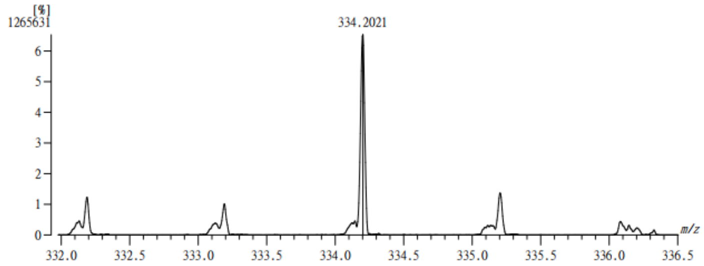

	Observed m/z	Int%	Err[ppm / mmu]	U.S.	Composition
1	334.2021	6.53	-40.8 / -13.6	11.0	C21 H26 N4
2			-3.2 / -1.1	11.5	C20 H24 N5
3			+34.4 / +11.5	12.0	C19 H22 N6
4			-44.9 / -15.0	10.5	C23 H28 N O
5			-7.2 / -2.4	11.0	C22 H26 N2 O
6			+30.4 / +10.2	11.5	C21 H24 N3 O
7			-24.8 / -8.3	7.5	C14 H24 N9 O
8			+12.8 / +4.3	8.0	C13 H22 N10 O
9			+26.4 / +8.8	11.0	C23 H26 O2
10			-28.8 / -9.6	7.0	C16 H26 N6 O2
11			+8.8 / +3.0	7.5	C15 H24 N7 O2
12			+46.5 / +15.5	8.0	C14 H22 N8 O2
13			-32.8 / -11.0	6.5	C18 H28 N3 O3
14			+4.8 / +1.6	7.0	C17 H26 N4 O3
15			+42.4 / +14.2	7.5	C16 H24 N5 O3
16			-50.4 / -16.8	3.0	C10 H26 N10 O3
17			-36.8 / -12.3	6.0	C20 H30 O4
18			+0.8 / +0.3	6.5	C19 H28 N O4
19			+38.4 / +12.8	7.0	C18 H26 N2 O4
20			-54.4 / -18.2	2.5	C12 H28 N7 O4
21			-16.8 / -5.6	3.0	C11 H26 N8 O4
22			+20.9 / +7.0	3.5	C10 H24 N9 O4
23			+58.5 / +19.6	4.0	C9 H22 N10 O4
24			-58.4 / -19.5	2.0	C14 H30 N4 O5
25			-20.8 / -6.9	2.5	C13 H28 N5 O5
26			+16.9 / +5.6	3.0	C12 H26 N6 O5
27			+54.5 / +18.2	3.5	C11 H24 N7 O5
28			-24.8 / -8.3	2.0	C15 H30 N2 O6
29			+12.8 / +4.3	2.5	C14 H28 N3 O6
30			+50.5 / +16.9	3.0	C13 H26 N4 O6
31			+8.8 / +2.9	2.0	C16 H30 O7
32			+46.4 / +15.5	2.5	C15 H28 N O7

Figure S38. HR-FAB-MS data of photoglaciapyrrole D (**4**)

[Mass Spectrum]
Data : FAB-B051 Date : 09-Sep-2020 15:57
RT : 0.37 min Scan# : (14,45)
Elements : C 100/0, H 100/0, N 10/0, O 10/0
Mass Tolerance : 1000ppm, 5mmu if m/z < 5, 20mmu if m/z > 20
Unsaturation (U.S.) : -0.5 - 20.0

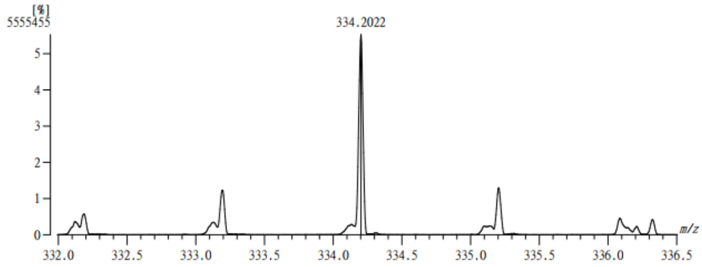

	Observed m/z	Int%	Err[ppm / mmu]	U.S.	Composition
1	334.2022	5.53	-40.5 / -13.5	11.0	C21 H26 N4
2			-2.9 / -1.0	11.5	C20 H24 N5
3			+34.7 / +11.6	12.0	C19 H22 N6
4			-44.6 / -14.9	10.5	C23 H28 N O
5			-6.9 / -2.3	11.0	C22 H26 N2 O
6			+30.7 / +10.3	11.5	C21 H24 N3 O
7			-24.5 / -8.2	7.5	C14 H24 N9 O
8			+13.1 / +4.4	8.0	C13 H22 N10 O
9			+26.7 / +8.9	11.0	C23 H26 O2
10			-28.5 / -9.5	7.0	C16 H26 N6 O2
11			+9.1 / +3.1	7.5	C15 H24 N7 O2
12			+46.8 / +15.6	8.0	C14 H22 N8 O2
13			-32.5 / -10.9	6.5	C18 H28 N3 O3
14			+5.1 / +1.7	7.0	C17 H26 N4 O3
15			+42.7 / +14.3	7.5	C16 H24 N5 O3
16			-50.1 / -16.7	3.0	C10 H26 N10 O3
17			-36.5 / -12.2	6.0	C20 H30 O4
18			+1.1 / +0.4	6.5	C19 H28 N O4
19			+38.7 / +12.9	7.0	C18 H26 N2 O4
20			-54.1 / -18.1	2.5	C12 H28 N7 O4
21			-16.5 / -5.5	3.0	C11 H26 N8 O4
22			+21.2 / +7.1	3.5	C10 H24 N9 O4
23			+58.8 / +19.7	4.0	C9 H22 N10 O4
24			-58.1 / -19.4	2.0	C14 H30 N4 O5
25			-20.5 / -6.8	2.5	C13 H28 N5 O5
26			+17.2 / +5.7	3.0	C12 H26 N6 O5
27			+54.8 / +18.3	3.5	C11 H24 N7 O5
28			-24.5 / -8.2	2.0	C15 H30 N2 O6
29			+13.1 / +4.4	2.5	C14 H28 N3 O6
30			+50.8 / +17.0	3.0	C13 H26 N4 O6
31			+9.1 / +3.0	2.0	C16 H30 O7
32			+46.7 / +15.6	2.5	C15 H28 N O7

Figure S39. HR-FAB-MS data of photoglaciapyrrole E (**5**)

[Mass Spectrum]
Data : FAB-B052 Date : 09-Sep-2020 16:04
RT : 1.30 min Scan# : (47,59)
Elements : C 100/0, H 100/0, N 10/0, O 10/0
Mass Tolerance : 1000ppm, 5mmu if m/z < 5, 20mmu if m/z > 20
Unsaturation (U.S.) : -0.5 - 20.0

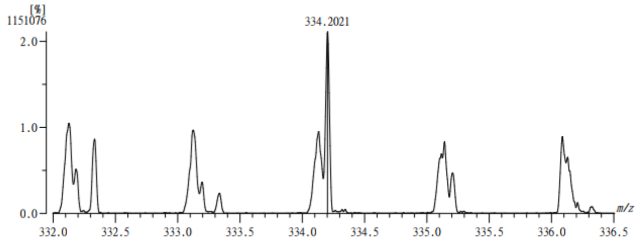

	Observed m/z	Int%	Err[ppm / mmu]	U.S.	Composition
1	334.2021	2.11	-40.8 / -13.6	11.0	C21 H26 N4
2			-3.2 / -1.1	11.5	C20 H24 N5
3			+34.4 / +11.5	12.0	C19 H22 N6
4			-44.9 / -15.0	10.5	C23 H28 N O
5			-7.2 / -2.4	11.0	C22 H26 N2 O
6			+30.4 / +10.2	11.5	C21 H24 N3 O
7			-24.8 / -8.3	7.5	C14 H24 N9 O
8			+12.8 / +4.3	8.0	C13 H22 N10 O
9			+26.4 / +8.8	11.0	C23 H26 O2
10			-28.8 / -9.6	7.0	C16 H26 N6 O2
11			+8.8 / +3.0	7.5	C15 H24 N7 O2
12			+46.5 / +15.5	8.0	C14 H22 N8 O2
13			-32.8 / -11.0	6.5	C18 H28 N3 O3
14			+4.8 / +1.6	7.0	C17 H26 N4 O3
15			+42.4 / +14.2	7.5	C16 H24 N5 O3
16			-50.4 / -16.8	3.0	C10 H26 N10 O3
17			-36.8 / -12.3	6.0	C20 H30 O4
18			+0.8 / +0.3	6.5	C19 H28 N O4
19			+38.4 / +12.8	7.0	C18 H26 N2 O4
20			-54.4 / -18.2	2.5	C12 H28 N7 O4
21			-16.8 / -5.6	3.0	C11 H26 N8 O4
22			+20.9 / +7.0	3.5	C10 H24 N9 O4
23			+58.5 / +19.6	4.0	C9 H22 N10 O4
24			-58.4 / -19.5	2.0	C14 H30 N4 O5
25			-20.8 / -6.9	2.5	C13 H28 N5 O5
26			+16.9 / +5.6	3.0	C12 H26 N6 O5
27			+54.5 / +18.2	3.5	C11 H24 N7 O5
28			-24.8 / -8.3	2.0	C15 H30 N2 O6
29			+12.8 / +4.3	2.5	C14 H28 N3 O6
30			+50.5 / +16.9	3.0	C13 H26 N4 O6
31			+8.8 / +2.9	2.0	C16 H30 O7
32			+46.4 / +15.5	2.5	C15 H28 N O7

Figure S40. HR-FAB-MS data of photoglaciapyrrole A (**6**)

[Mass Spectrum]
Data : FAB-B050 Date : 09-Sep-2020 15:48
RT : 0.54 min Scan# : (20,22)
Elements : C 100/0, H 100/0, N 10/0, O 10/0
Mass Tolerance : 1000ppm, 5mmu if m/z < 5, 20mmu if m/z > 20
Unsaturation (U.S.) : 0.0 - 20.0

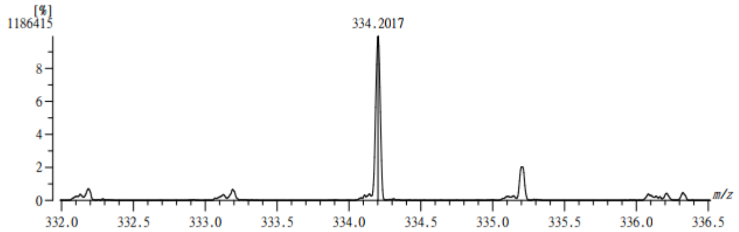

	Observed m/z	Int%	Err[ppm / mmu]	U.S.	Composition
1	334.2017	9.94	-42.0 / -14.0	11.0	C21 H26 N4
2			-4.4 / -1.5	11.5	C20 H24 N5
3			+33.2 / +11.1	12.0	C19 H22 N6
4			-46.0 / -15.4	10.5	C23 H28 N O
5			-8.4 / -2.8	11.0	C22 H26 N2 O
6			+29.2 / +9.8	11.5	C21 H24 N3 O
7			-26.0 / -8.7	7.5	C14 H24 N9 O
8			+11.7 / +3.9	8.0	C13 H22 N10 O
9			+25.2 / +8.4	11.0	C23 H26 O2
10			-30.0 / -10.0	7.0	C16 H26 N6 O2
11			+7.6 / +2.6	7.5	C15 H24 N7 O2
12			+45.3 / +15.1	8.0	C14 H22 N8 O2
13			-34.0 / -11.4	6.5	C18 H28 N3 O3
14			+3.6 / +1.2	7.0	C17 H26 N4 O3
15			+41.2 / +13.8	7.5	C16 H24 N5 O3
16			-51.6 / -17.2	3.0	C10 H26 N10 O3
17			-38.0 / -12.7	6.0	C20 H30 O4
18			-0.4 / -0.1	6.5	C19 H28 N O4
19			+37.2 / +12.4	7.0	C18 H26 N2 O4
20			-55.6 / -18.6	2.5	C12 H28 N7 O4
21			-18.0 / -6.0	3.0	C11 H26 N8 O4
22			+19.7 / +6.6	3.5	C10 H24 N9 O4
23			+57.3 / +19.2	4.0	C9 H22 N10 O4
24			-59.6 / -19.9	2.0	C14 H30 N4 O5
25			-22.0 / -7.3	2.5	C13 H28 N5 O5
26			+15.7 / +5.2	3.0	C12 H26 N6 O5
27			+53.3 / +17.8	3.5	C11 H24 N7 O5
28			-26.0 / -8.7	2.0	C15 H30 N2 O6
29			+11.6 / +3.9	2.5	C14 H28 N3 O6
30			+49.3 / +16.5	3.0	C13 H26 N4 O6
31			+7.6 / +2.5	2.0	C16 H30 O7
32			+45.2 / +15.1	2.5	C15 H28 N O7

Figure S41. Comparison of the experimental and calculated ECD spectra of glaciapyrrole D (**1**)

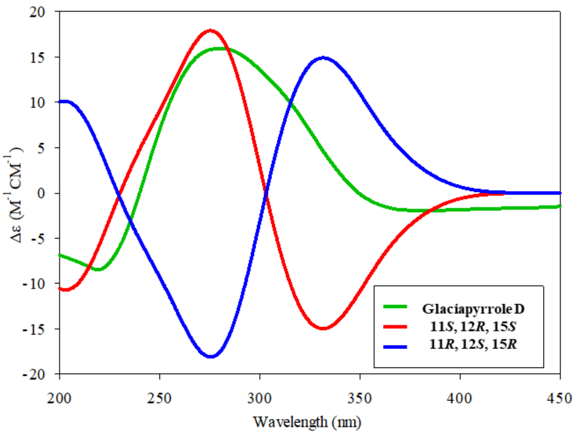

Figure S42. ^{1}H NMR signal intensity plot of photoglaciapyrrole A and glaciapyrrole A according to UV (366 nm) irradiation time when purified photoglaciapyrrole was irradiated. Each point is an integration value (an arbitrary unit) of ^{1}H NMR signals at H-9 position of corresponding compounds.

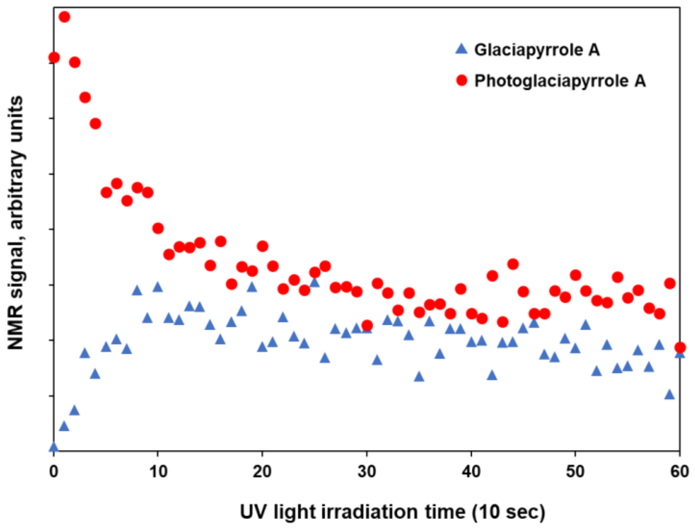

Figure S43. A proposed reversible mechanism for the double bond photoisomerism.

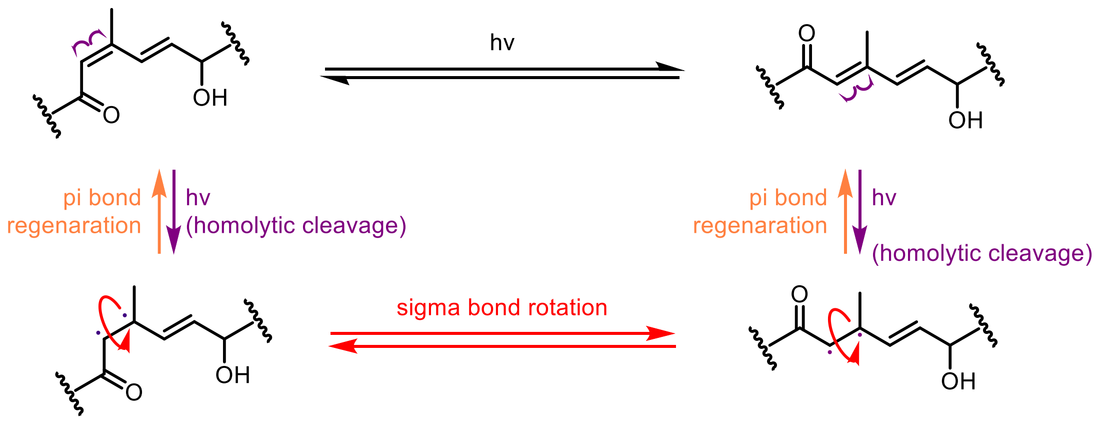

Table S1. Cartesian coordinates of glaciapyrrole D (**1**)

No.	atom	Cartesian coordinates		
1	C	0.96932135084961	-5.61460885106199	1.12766072651951
2	C	1.04952493729919	-8.24531280094804	1.06212453444250
3	C	3.39207112768224	-4.73541664858595	0.40936597877594
4	C	4.89499821614879	-6.85528617501927	-0.07942600560488
5	N	3.40954070441186	-8.94944145890722	0.33720343335780
6	H	-0.66499973163303	-4.46961736932586	1.63888062553236
7	H	-0.40900304689316	-9.63755278226243	1.48234981404169
8	H	3.98183409302655	-2.76882589923946	0.26361398505318
9	C	7.52536879793155	-7.27879478987752	-0.88555230529162
10	O	8.26130681033872	-9.47819106250595	-1.14475269872130
11	C	9.07076710096588	-4.99400996291127	-1.32167360049907
12	C	11.54159385591278	-4.85385406039956	-2.05787133657744
13	H	8.07814518580659	-3.20628082503334	-1.01685483239013
14	C	12.73638448563917	-2.28520453833520	-2.42318672006109
15	H	11.39739216806139	-0.74749510035792	-1.99698629074190
16	H	13.39842370582684	-2.03823442284213	-4.39044735128102
17	H	14.40991715854982	-2.05253383549662	-1.19342589817335
18	C	13.08233169991428	-7.10273284043860	-2.51699157270412
19	C	15.48701066259627	-7.07853072276492	-3.35997336454322
20	H	12.17343016850981	-8.91470582237521	-2.15095260271291
21	C	17.00260833342658	-9.42290934434609	-3.86705266486455
22	H	16.49816765175353	-5.32119002129927	-3.77375238710533
23	H	4.10579636050773	-10.72176523079488	0.10100515878806
24	O	15.67807189234031	-11.57448718347319	-3.01816515699599
25	C	19.73792205578110	-9.33233012328152	-2.74694271388809
26	H	17.26568614812913	-9.56353007228683	-5.96024030962895
27	C	21.03278022184694	-11.86158706966780	-3.39895078877584
28	C	19.43990859142302	-14.16638737758696	-2.64659369614851
29	H	22.92018855523571	-11.91995090396591	-2.50863720574618
30	H	21.34591024262572	-11.90199134944655	-5.47332138854095
31	C	16.76469569743993	-13.92185748294825	-3.72864488355384
32	H	19.34470697740708	-14.30934822314878	-0.56895557624601
33	H	20.30714332605456	-15.92996457708791	-3.33863768539612
34	O	21.08429868747662	-7.27960995321047	-3.85947574518610
35	C	19.70820996953827	-8.81592703264962	0.09336956159247
36	H	18.47156967211926	-10.15992433734439	1.09406377703173
37	H	19.00434420074620	-6.88934513220549	0.46203126879528
38	H	21.64102345990228	-8.95806017920458	0.86704003525333
39	H	21.41637773653345	-7.68909461944443	-5.62084224620473
40	C	14.89375782568925	-16.06415243121184	-2.99573267697676
41	H	16.91899736605682	-13.96499355173732	-5.83144144856026
42	C	12.33681464194023	-15.64194299481193	-4.30107552369166
43	H	11.38200446985369	-13.92618381635404	-3.60379636640411
44	H	12.58642520761713	-15.43730403590606	-6.36993160290469
45	H	11.10248642000718	-17.28867677688357	-3.95426335974419
46	C	14.53949190547866	-16.29752108799655	-0.13665458965494
47	H	13.08561150644289	-17.73519322752152	0.27540692333436
48	H	13.93609376725062	-14.47617994733949	0.67562067638090
49	H	16.30604159601510	-16.90944342177173	0.78546625431803
50	O	15.97209879266044	-18.40804387584918	-3.81307118913745
51	H	15.86328952457206	-18.47818055265714	-5.64644397844336

Table S2. Cartesian coordinates of *ent*-glaciapyrrole D (**1**)

No.	atom	Cartesian coordinates		
1	C	2.25664393778993	-3.74205025139863	-0.54481587673106
2	C	2.92721237200253	-6.26376845009227	-0.19410629011241
3	C	4.46004751998871	-2.27841505645686	-0.14190520519636
4	C	6.42612002596234	-3.94597598618377	0.44619340843299
5	N	5.42428551341945	-6.34588767776241	0.39656458745777
6	H	9.12849760857656	-3.68829652240720	1.06092415725246
7	H	10.35292694172995	-5.62648936069191	1.49654749087811
8	H	10.14616595965486	-1.08835642509331	1.10661515951867
9	C	12.56302683914200	-0.32895035078369	1.60480526175425
10	O	13.17255586415374	2.46146039032447	1.58184886929990
11	C	14.67485192073952	2.89129384708425	0.19443850595323
12	C	13.87877187303410	3.09156561263876	3.44526315622878
13	H	11.49994450786491	3.60881069279797	1.10651013042904
14	C	8.76135905337719	0.39076703349252	0.69520291652925
15	H	14.59406645362797	-2.11782885149552	2.16858694088742
16	H	16.97112771537259	-1.46973266640396	2.81765662301392
17	H	14.10005570195966	-4.11485703366072	2.06143452172160
18	C	19.00404781125247	-3.34929489076749	3.43790224376312
19	C	17.57639000822748	0.50264240046879	2.97737641757513
20	H	18.17583703261371	-5.82070715815372	2.88204157514243
21	C	21.59536870134579	-2.76575569925970	2.13683226725180
22	H	19.38206724539159	-3.20598144368226	5.51357827426385
23	H	22.47667539455797	-0.35502136860752	2.95675626400471
24	O	21.32904968873359	-2.58151232246704	-0.73207418031230
25	C	20.17497358545990	-0.92130270264308	-1.23740092969711
26	H	20.40599808001851	-4.27529007401544	-1.51597044833415
27	C	23.20812962526553	-2.35756040687740	-1.61113877326089
28	C	23.46469262011041	-4.85360970731457	2.94466108003068
29	H	22.41576210173645	-7.52567641379692	2.52595794888742
30	H	23.86493716993158	-4.59539883551106	4.98846037635664
31	C	25.27675714982016	-4.58425557983039	1.94288743758279
32	H	19.80087065628647	-7.77320084819656	3.74566843284322
33	H	23.69289700209472	-8.96233817930630	3.32969962264034
34	O	22.27381813780158	-7.91475244974500	0.48280937238153
35	C	18.44713334585970	-10.34792330682322	3.34096928649450
36	H	20.04330352616684	-7.55249333566456	5.82942226744089
37	H	15.91532205223094	-10.37403112623208	4.75936799079508
38	H	14.56535956093426	-8.99960156430771	3.96574984702956
39	H	15.07862760992736	-12.28297269916403	4.64830383881085
40	C	16.19483407880352	-9.90018386200787	6.77954410315522
41	H	18.04375269659275	-10.96314214030427	0.54579927084003
42	C	16.99932650448196	-9.42690549560397	-0.39706772339944
43	H	16.95189552465900	-12.73120342368689	0.36796956027226
44	H	19.86601315368477	-11.24809468644476	-0.42513834986484
45	H	20.06901493170117	-12.28676723037806	4.30895846523045
46	C	6.51266562531095	-7.88221956892973	0.76644228554026
47	H	0.37992566542561	-3.04946738661432	-1.03642428216553
48	H	1.79433601289625	-7.97863912160578	-0.32696097621597
49	H	4.60172108154654	-0.22868853471974	-0.26670674025774
50	O	22.96676727666866	-0.48553114800460	4.72388717478719
51	H	20.06053112244159	-12.17547178137035	6.14355747098986

Table S3. ECD calculations of glaciapyrrole D (**1**)
(a) ECD calculations of glaciapyrrole D (**1**).
total energy = -1094.363684814
kinetic energy = 1084.304734068
potential energy = -2178.668418882

Parameters of Level DFT
DFT settings (Functional B3LYP / Gridsize M3)
Geometry optimization options (Energy 10-6 Hartree, Gradient norm │dE / dxyz│= 10-3 Hartree/Bohr)

Energy minimized coordinates of glaciapyrrole D at the basis set def2-TVZPP for all atoms (Å).

atomic coordinates			atom	charge
0.96721532	-5.61715159	1.12096379	C	6
1.04924683	-8.24784632	1.05690747	C	6
3.39018209	-4.73671587	0.40475710	C	6
4.89506851	-6.85587058	-0.08126102	C	6
3.41053213	-8.95077226	0.33490791	N	7
-0.66839739	-4.47297969	1.62992311	H	1
-0.40882924	-9.64083173	1.47625383	H	1
3.97873704	-2.76979183	0.25842178	H	1
7.52643982	-7.27817347	-0.88477536	C	6
8.26397996	-9.47724732	-1.14214249	O	8
9.07098793	-4.99279269	-1.32067237	C	6
11.54202348	-4.85221483	-2.05600489	C	6
8.07763389	-3.20532227	-1.01660776	H	1
12.73705250	-2.28349525	-2.42010040	C	6
11.39793456	-0.74586356	-1.99401079	H	1
13.39991725	-2.03598604	-4.38701245	H	1
14.41007539	-2.05122865	-1.18956775	H	1
13.08271079	-7.10115067	-2.51482494	C	6
15.48729339	-7.07744825	-3.35808901	C	6
12.17369697	-8.91299898	-2.14837505	H	1
17.00233174	-9.42235074	-3.86470443	C	6
16.49865831	-5.32037959	-3.77251673	H	1
4.10819352	-10.72276303	0.10038056	H	1
15.67797569	-11.57403729	-3.01597749	O	8
19.73877009	-9.33278974	-2.74747594	C	6
17.26404946	-9.56325523	-5.95800107	H	1
21.03208553	-11.86190857	-3.40337785	C	6
19.43946476	-14.16688049	-2.65051328	C	6
22.92084993	-11.92155352	-2.51607367	H	1
21.34194494	-11.90074779	-5.47829398	H	1
16.76296501	-13.92127113	-3.72928140	C	6
19.34657821	-14.31092642	-0.57280698	H	1
20.30559238	-15.93024953	-3.34443570	H	1
21.08425812	-7.27946877	-3.85983733	O	8
19.71241267	-8.81914808	0.09339963	C	6
18.47669780	-10.16394360	1.09415874	H	1
19.00935560	-6.89281048	0.46485073	H	1
21.64609103	-8.96244704	0.86467446	H	1
21.41415441	-7.68755295	-5.62192651	H	1
14.89267044	-16.06321108	-2.99377917	C	6
16.91421229	-13.96382225	-5.83232923	H	1
12.33371228	-15.64010454	-4.29488124	C	6
11.38051913	-13.92420358	-3.59574697	H	1
12.57997013	-15.43519703	-6.36411085	H	1
11.09951092	-17.28655044	-3.94629966	H	1

14.54303983	-16.29685422	-0.13416084	C	6
13.08945763	-17.73418447	0.28013049	H	1
13.94149683	-14.47545586	0.67935128	H	1
16.31096017	-16.90936930	0.78494787	H	1
15.96909860	-18.40726777	-3.81318729	O	8
15.85730360	-18.47711953	-5.64638190	H	1

(b) ECD calculations of *ent*-glaciapyrrole D (**1**).
total energy = -1094.363688077
kinetic energy = 1084.303769185
potential energy = -2178.667457262

Parameters of Level DFT
DFT settings (Functional B3LYP / Gridsize M3)
Geometry optimization options (Energy 10-6 Hartree, Gradient norm │dE / dxyz│= 10-3 Hartree/Bohr)

Energy minimized coordinates of *ent*-glaciapyrrole D at the basis set def2-TVZPP for all atoms (Å).

atomic coordinates			atom	charge
2.25979306	-3.74511852	-0.5497837	C	6
2.93100422	-6.26646811	-0.19761984	C	6
4.46224210	-2.28050228	-0.14507916	C	6
6.42835383	-3.94714441	0.44557737	C	6
5.42742389	-6.34740894	0.39588549	N	7
9.13025769	-3.68846193	1.06216235	C	6
10.35483420	-5.62608365	1.49942043	O	8
10.14726925	-1.08821656	1.10759589	C	6
12.56385130	-0.32827968	1.60644093	C	6
13.17293630	2.46224451	1.58361469	C	6
14.67558991	2.89234009	0.19667239	H	1
13.87848119	3.09247656	3.44724651	H	1
11.50026198	3.60930703	1.10776865	H	1
8.76223139	0.39056010	0.69564218	H	1
14.59492545	-2.11694750	2.17079400	C	6
16.97209111	-1.46889817	2.81947938	C	6
14.10083237	-4.11397610	2.06420337	H	1
19.00467848	-3.34875526	3.43977486	C	6
17.57764391	0.50341339	2.97881715	H	1
18.17515786	-5.81970414	2.88385141	O	8
21.59530219	-2.76591093	2.13683928	C	6
19.38359828	-3.20535084	5.51531389	H	1
22.47852454	-0.35626376	2.95779101	O	8
21.32655444	-2.57920567	-0.73172934	C	6
20.17294229	-0.91794833	-1.23464310	H	1
20.40185673	-4.27182647	-1.51615168	H	1
23.20494438	-2.35556125	-1.61235065	H	1
23.46445210	-4.85514479	2.94153037	C	6
22.41394673	-7.52628639	2.52147392	C	6
23.86660833	-4.59872986	4.98517623	H	1
25.27572155	-4.58580715	1.93832834	H	1
19.80062260	-7.77317561	3.74440269	C	6
23.69136139	-8.96432501	3.32230140	H	1
22.26911098	-7.91332668	0.47816167	H	1
18.44520169	-10.34727535	3.34170114	C	6
20.04611023	-7.55293541	5.82783517	H	1
15.91567910	-10.37222069	4.76415951	C	6

14.56503299	-8.99721254	3.97274185	H	1
15.07796985	-12.28078769	4.65447776	H	1
16.19866997	-9.89846547	6.78387397	H	1
18.03713715	-10.96231786	0.54718102	C	6
16.99198724	-9.42559067	-0.39405900	H	1
16.94411590	-12.72982489	0.37109357	H	1
19.85773056	-11.24821226	-0.42659736	H	1
20.06777966	-12.28678048	4.30720989	O	8
6.51586060	-7.88317974	0.76788457	H	1
0.38326498	-3.05339861	-1.04338006	H	1
1.79904098	-7.98186184	-0.33150106	H	1
4.60323646	-0.23073301	-0.27029034	H	1
22.97002282	-0.48835696	4.72442029	H	1
20.06221537	-12.17542336	6.14182559	H	1
